# Complementary *Operando* Spectroscopy identification of in-situ generated metastable charge-asymmetry Cu_2_-CuN_3_ clusters for CO_2_ reduction to ethanol

**DOI:** 10.1038/s41467-022-29035-8

**Published:** 2022-03-11

**Authors:** Xiaozhi Su, Zhuoli Jiang, Jing Zhou, Hengjie Liu, Danni Zhou, Huishan Shang, Xingming Ni, Zheng Peng, Fan Yang, Wenxing Chen, Zeming Qi, Dingsheng Wang, Yu Wang

**Affiliations:** 1 Shanghai Synchrotron Radiation Facility, Zhangjiang Laboratory, Shanghai Advanced Research Institute, Chinese Academy of Sciences, Shanghai, 201204 China; 2 Beijing Key Laboratory of Construction Tailorable Advanced Functional Materials and Green Applications, School of Materials Science and Engineering, Beijing Institute of Technology, Beijing, 100081 China; 3 Department of Chemistry, Tsinghua University, Beijing, 100084 China; 4 Shanghai Institute of Applied Physics, Chinese Academy of Sciences, Shanghai, 201800 China; 5 National Synchrotron Radiation Laboratory, University of Science and Technology of China, Hefei, Anhui 230029 China; 6 School of Physical Science and Technology, ShanghaiTech University, Shanghai, 201210 China

**Keywords:** Electrocatalysis, Energy

## Abstract

Copper-based materials can reliably convert carbon dioxide into multi-carbon products but they suffer from poor activity and product selectivity. The atomic structure-activity relationship of electrocatalysts for the selectivity is controversial due to the lacking of systemic multiple dimensions for *operando* condition study. Herein, we synthesized high-performance CO_2_RR catalyst comprising of CuO clusters supported on N-doped carbon nanosheets, which exhibited high C_2+_ products Faradaic efficiency of 73% including decent ethanol selectivity of 51% with a partial current density of 14.4 mA/cm^−2^ at −1.1 V vs. RHE. We evidenced catalyst restructuring and tracked the variation of the active states under reaction conditions, presenting the atomic structure-activity relationship of this catalyst. *Operando* XAS, XANES simulations and Quasi-in-situ XPS analyses identified a reversible potential-dependent transformation from dispersed CuO clusters to Cu_2_-CuN_3_ clusters which are the optimal sites. This cluster can’t exist without the applied potential. The N-doping dispersed the reduced Cu_n_ clusters uniformly and maintained excellent stability and high activity with adjusting the charge distribution between the Cu atoms and N-doped carbon interface. By combining *Operando* FTIR and DFT calculations, it was recognized that the Cu_2_-CuN_3_ clusters displayed charge-asymmetric sites which were intensified by CH_3_^*^ adsorbing, beneficial to the formation of the high-efficiency asymmetric ethanol.

## Abstract (web summary)

Copper-based materials can convert carbon dioxide into multi-carbon products but suffer from poor activity and selectivity. Here, the authors report CuO clusters supported on nitrogen-doped carbon nanosheets for the reduction CO_2_-to-ethanol, and investigate the change in the catalytic sites while in operation.

## Introduction

Electrochemical conversion of carbon dioxide into value-added products using sustainable energies is environmentally friendly and economical approach^1^. As well known, C_2+_ hydrocarbons or oxygenated compounds such as ethanol (C_2_H_5_OH), ethylene (C_2_H_4_) and n-propanol (n-C_3_H_7_OH) with remarkable energy densities have more value for solving the energy crisis^2^. However, complex product selectivity limits the development and application of carbon dioxide reduction. The origin of this selectivity toward C_2+_ hydrocarbons has been a prominent topic of study recently and has been intensely debated^3–6^. Copper-based materials are absolutely effective and promising catalysts for the reduction of carbon dioxide to multi-carbon products^3^. Previous works indicated that both the catalysts and substrates could affect the local structure and the electronic state of active sites, and then modify the reaction pathway and catalytic mechanism^4,5^. Many strategies have been developed to modify or optimize the copper-based catalysts for improving the multi-carbon products selectivity, such as adjusting the surface morphology^4,5,7–13^, doping metals or non-metals^14–18^, building nanostructures^19–21^, or designing single active sites^22–25^.

Despite tremendous effort has been paid, further advances are still needed to understand the structure-activity relationship (SAR) and efficient conversion CO_2_ to multi-carbon products especially for high economy asymmetric C_2+_ products (e.g., ethanol). Therefore, the design of catalysts that selectively produce ethanol via electrochemical CO_2_RR were supposed to focus on minimizing three competing reaction pathways which reduced the faradaic efficiency (FE) of asymmetric ethanol by consuming electrons and protons, the hydrogen evolution reaction (HER), C_1_ product formation (e.g., CH_4_, HCOOH) and other symmetrical C_2_ product formation (e.g., ethylene). However, little is known about the structure-activity relationship, extraordinarily to explain the catalyst structure changes with the product changes under different potential. Hence, it is urgent to build the structure-activity relationship between atomic structure and products for designing electrocatalysts.

Here we synthesized CuO clusters supported on nitrogen-doped carbon nanosheets (Cu/N_0.14_C) as dispersed electrocatalysts, achieving high-performances CO_2_ reduction that are superior to CuO_x_ catalysts in terms of stability, activity and selectivity. The prepared Cu/N_0.14_C catalyst with appropriate nitrogen content displayed high C_2+_ products Faradaic efficiency of 73% which includes ethanol FE of 51% at the potential of −1.1 V vs. RHE with a current density of −14.4 mA/cm^−2^ in 0.1 M KHCO_3_ electrolyte. Moreover, it exhibited superb long-term CO_2_ electroreduction durability over 10 h. The well-defined self-reconstruction for the active sites of Cu/N_0.14_C also facilitate the in-depth mechanistic understandings from complementary *Operando* Spectroscopy (XAS, XPS and FTIR), XANES simulations and theoretical calculations to present the atomic structure-activity relationship between the ethanol selectivity and in situ dynamic structure (local structure, electronic structure and the adsorbed intermediates), that would benefit the recognition for the CO_2_RR process.

## Results and discussion

### Structure characterization of the Cu/N_x_C

**Fig. 1:**
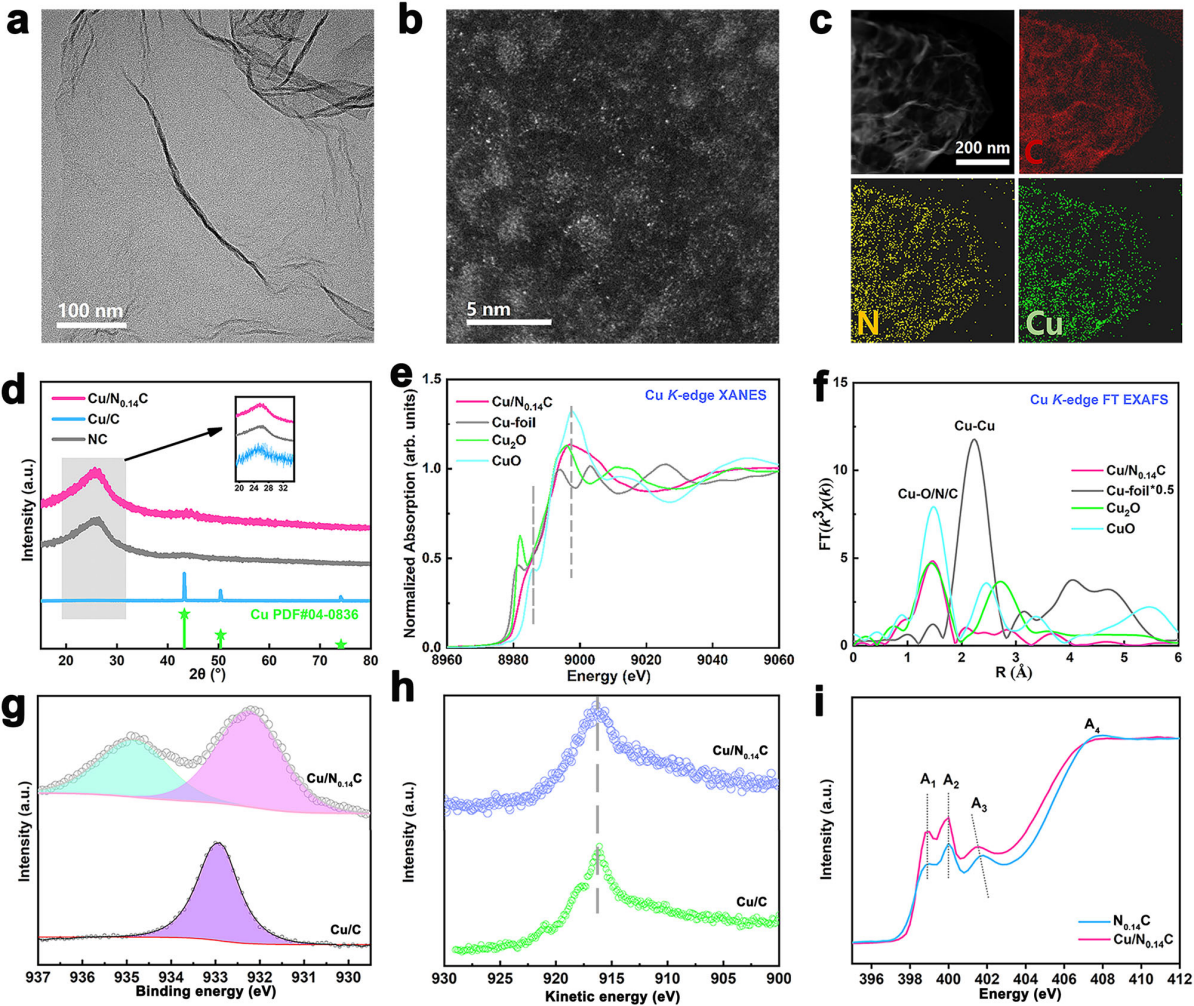
Structure characterizations of the Cu/N_x_C. **a** TEM image of Cu/N_0.14_C. **b** HAADF-STEM image of Cu/N_0.14_C. **c** EDS of Cu/N_0.14_C. **d** XRD of Cu/N_0.14_C, Cu/C and NC. **e** Cu K-edge XANES spectra of Cu/N_0.14_C with the references. **f** FT-EXAFS spectra of Cu/N_0.14_C with the references. **g** XPS analysis of the Cu 2p_3/2_ for Cu/N_0.14_C and Cu/C. **h** AES analysis of the Cu L3M45M45 for Cu/N_0.14_C and Cu/C. **i** N K-edge XANES spectra of Cu/N_0.14_C and N_0.14_C.

In a typical procedure, copper phthalocyanine and dicyandiamide were continuously mixed in ethanol and dried. Subsequently, the dried mixture was pyrolyzed at different temperatures for a series of Cu/N_x_C samples with different nitrogen content (x = 0.14, 0.11, 0.02, 0, x is mass contents ratio of nitrogen to carbon). The linear sweep voltammetry (LSV) curves demonstrated that the activity of Cu/N_0.14_C was better than other contrast samples with maximum current density. Transmission electron microscopy (TEM) revealed that Cu/N_x_C displayed a nano flake structure without obvious large-scale particles on the surface (Fig. 1a and Supplementary Fig. 1). The nanosheet structure was further confirmed by atomic force microscopy (AFM), and the corresponding height profiles of the scans showed a thickness of about 0.6 nm (Supplementary Fig. 2). The N species in the samples could prevent Cu atoms from aggregating excessively and ensured the atomic dispersion of Cu species. As we found in Supplementary Fig. 3, Cu nanoparticles (about 2–3 nano) were observed in the sample Cu/C (without N). High angle annular dark-field scanning transmission electron microscopy (HAADF-STEM), which can be clearly observed in the atomic phase, revealed that the copper atoms of Cu/N_0.14_C agglomerate into clusters without an obvious lattice (Fig. 1b). Elemental mapping by Energy Dispersive Spectrometer (EDS) (Fig. 1c) demonstrates that nitrogen is uniformly dispersed within the carbon matrix. X-ray diffraction (XRD) revealed only a broad peak from the (002) of the Cu/N_0.14_C and N_0.14_C (Fig. 1d), however the Cu/C showed totally different peaks which could belong to a pure metallic copper (PDF#04-0836). The electron diffraction pattern of Cu/N_0.14_C revealed it was amorphous material in Supplementary Fig. 4. To further study the electronic structure of the catalysis, Cu K-edge X-ray absorption spectroscopy, X-ray photoelectron spectroscopy (XPS) and soft-X-ray absorption spectroscopy (XAS) were carried out as shown in Fig. 1e–g and i and Supplementary Fig. 5. The Cu 2p_3/2_ XPS fitting curves revealed that Cu was mainly +1 and +2 valence in Cu/N_0.14_C^26,27^. The Cu L3M45M45 Auger electron spectroscopy (AES) showed no distinct peak around 918.5 eV which indicated that no Cu^0^ existed on the surface. The N K-edge presented four obvious resonances (Fig. 1i) assigned peak A_1_, A_2_, and A_3_ to pyridine-like, cyanide-like, and graphite-like N, respectively, whereas A_4_ at ∼407 eV is attributed to the transition from N 1*s* core states to N−C σ* bonds^28^. Obviously, peak A_3_ of Cu/N_0.14_C shifts negative energy which indicated the charge transfer had occurred compared with that of N_0.14_C while other peaks keep the same position, indicating graphite-N formed a chemical bond with Cu sites^29^. The C K-edge XAS spectra in Supplementary Fig. 5 showed no difference between Cu/N_0.14_C and N_0.14_C, excluding formation of the Cu−C bond. Thus, we could conclude that Cu sites only bond to graphite-like N, in Cu/N_0.14_C.

X-ray absorption spectroscopy (XAS) measurements were employed to further investigate the electronic structure and geometric structure of the Cu/N_0.14_C. Fig. 1e showed the Cu K-edge XANES spectra of the Cu/N_0.14_C together with the references of CuO, Cu_2_O and Cu foil. The absorption edge of the Cu/N_0.14_C was between the Cu_2_O and CuO, indicating that the oxidation state of the copper species is in the intermediate valence state between +1 and +2. The feature of XANES at 8986 eV and 8997 eV for Cu/N_0.14_C resembled the mixture dominated by CuO with a little contribution by Cu^+^ or Cu^0^ complex. The Fourier-transformed (FT) *k*^3^-weighted EXAFS oscillation in Fig. 1f also showed the disappearance of the feature for Cu–Cu in R space in Cu/N_0.14_C with simultaneous appearance of the peak at 1.5 Å for Cu−O/N. The fitting result displayed that the Cu−O/N bond length is 1.93 Å with the coordination number (CN) 3.4, (Supplementary Figs. 6, 7 and Table 1) indicating that Cu ions in the as-synthesized catalysts were predominately in the form of CuO clusters coordinated with four oxygen atoms. Notably, the coordination number of Cu−O/N is lower than that of the CuO (CN = 4) with a litter shorter bond length (1.93 Å) (the bond length in CuO is 1.95 Å) suggesting that Cu species with a definite Cu–O/N coordination-unsaturated structure (CN < 4, bond length <1.95 Å) existed. Note that TEM and EDX results showed CuO clusters were uniformly dispersed and anchored on the N-doped carbon nanosheets support. To further explore the N-doped that influenced CO_2_RR activity, XANES and FT-EXAFS of Cu/C without the N-doping was plot in Supplementary Fig. 8. The XANES of the Cu/C indicated that the Cu ions were reduced to metallic Cu. The FT-EXAFS data also showed the peak for Cu–Cu in R space with the weak peak at 1.5 Å for Cu–O revealing that the copper in Cu/C agglomerated into larger metallic particles with a copper oxides surface in the air (more detail in Supplementary Figs. 9, 10 and Table 2). Our study indicated that N-doped promotes the dispersion of CuO clusters in the as-synthesized catalysts and played the critical role in improving FE for direct CO_2_-to-ethanol electrochemical conversion. These results agreed well with XRD and HAADF–STEM results. The structure of the Cu/N_0.14_C is further discussed by *operando* XAS.

### Electrochemical characterization

**Fig. 2:**
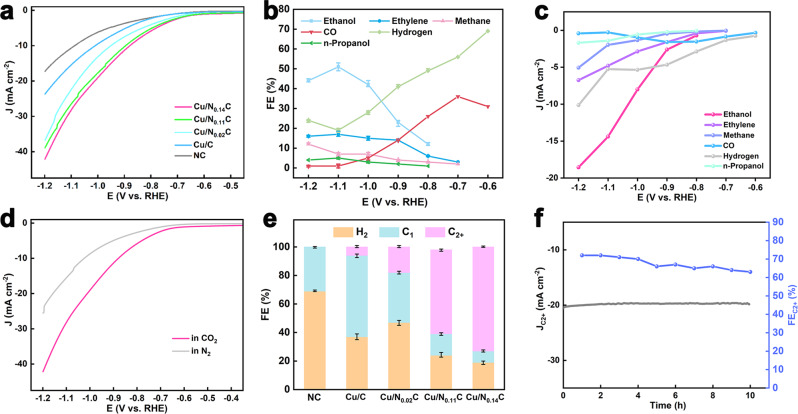
CO_2_RR activity of Cu/N_x_C. **a** LSV curves of N_0.14_C and Cu/N_x_C. **b** FE and **c** Partial current density of each product of Cu/N_0.14_C. **d** LSV curves of Cu/N_0.14_C in N_2_ and CO_2_. **e** FE of H_2_, C_1_ and C_2+_ products of N_0.14_C and Cu/N_x_C. **f** Long-time stability of Cu/N_0.14_C.

The electrochemical CO_2_RR activity of Cu/N_x_C catalysts were evaluated in CO_2_-saturated 0.1 M KHCO_3_ solution. Linear sweep voltammetry (LSV) curves (Fig. 2a) demonstrated that the activity of Cu/N_0.14_C was better than other contrast samples with lowest onset potential and maximum current density. This suggested that the increase in nitrogen content is helpful but limited to improve the activity. Versus Cu/N_0.14_C catalysts, the NC catalyst has little activity. Fig. 2b showed the product selectivity of CO_2_RR of Cu/N_0.14_C catalyst. The main products formed at low potential were CO and H_2_. At −0.7 V (versus the reversible hydrogen electrode, RHE), hydrocarbons such as ethylene (C_2_H_4_) and methane (CH_4_) started to form, and the selectivity of CO and H_2_ decreased. The product of ethanol (C_2_H_5_OH) was first detected at −0.8 V vs. RHE. Subsequently, the faradaic efficiency (FE) of C_2+_ products continuously increased in the range −0.8 V to −1.1 V. At −1.1 V, the maximum FE of ethanol is 51% with a current density of −14.4 mA/cm^−2^ and the total C_2+_ products FE is up to 73%. We also gave the each product partial current density of Cu/N_0.14_C catalyst in Fig. 2c. The LSV curves of Cu/N_0.14_C in N_2_ afignd CO_2_ were measured, respectively, in Fig. 2d. In CO_2_-saturated 0.1 M KHCO_3_ solution, the Cu/N_0.14_C displayed high activity. Fig. 2e showed that H_2_, C_1_ and C_2+_ products distributions of five catalysts. Cu/N_x_C catalysts truly boosted the product selectivity of CO_2_RR. We further evaluated the stability of Cu/N_0.14_C for CO_2_RR by a long-term experiment of 10 h. Both the partial current density and FE of C_2+_ products lightly decreased (Fig. 2f), that demonstrated good stability. There were no structural changes of Cu/N_0.14_C after long-term CO_2_RR electrolysis, as shown in Supplementary Fig. 11.

### *Operando* XAS, SR-FTIR Spectroscopy and Quasi-in-situ XPS-AES results

**Fig. 3:**
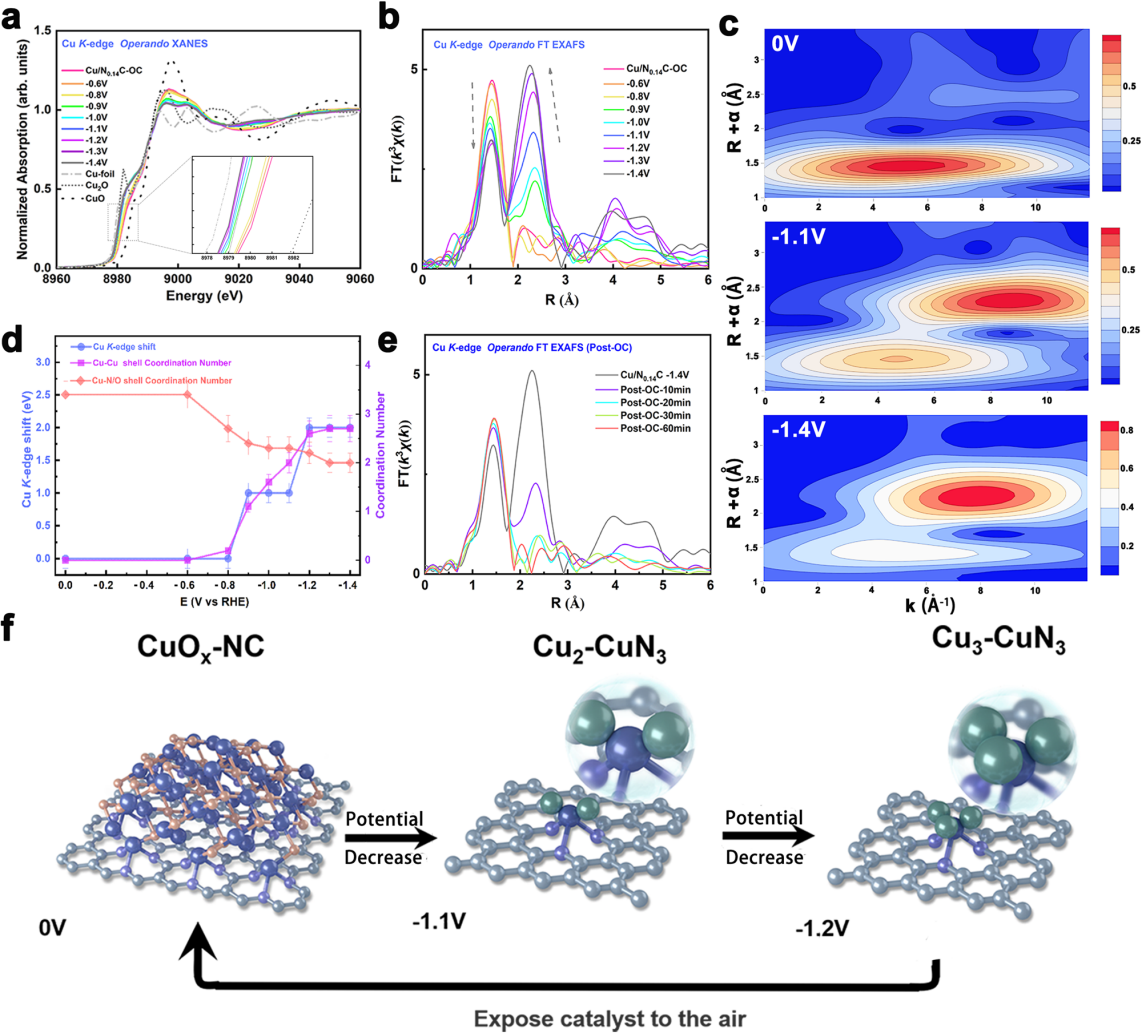
*Operando* XAFS characterization of Cu/N_0.14_C. **a** Cu K-edge *operando* XANES of Cu/N_0.14_C from OC to −1.4 V vs. RHE in 0.1 M KHCO_3._ **b** *Operando* FT-EXAFS of Cu/N_0.14_C. **c** Wavelet transforms for the *k*^*3*^-weighted Cu K-edge EXAFS signals at OC, −1.1 V and −1.4 V vs. RHE during the CO_2_RR. **d** The relationship of Cu/N_0.14_C absorption edge, CN of first shell, CN of second shell and the potential. **e** *Operando* FT-EXAFS of Cu/N_0.14_C after the applied potential was switched off and other conditions remained unchanged. **f** Proposed scheme for the reversible formation of the catalytically active Cu_n_–CuN_3_ cluster based on *operando* XAS and Quasi-in-situ XPS analysis (rufous, O; gray, C; purple, N; blue, Cu bond to both N and Cu; green, Cu just bond to Cu). All potentials are normalized to RHE.

Studying the atomic structure-activity relationship of CO_2_RR catalyst under *operando* conditions is crucial to reveal the intrinsic reactive mechanism. In order to identify the realistic catalytic centers of Cu/N_0.14_C, *operando* XAS study were employed to directly monitor its catalytic behavior. We first collected ex-situ XAS data of the fully activated Cu/N_0.14_C. When Cu/N_0.14_C was soaked into the KHCO_3_ solution at open circuit (OC), no obvious structural change was observed from the XANES in Supplementary Fig. 12. *Operando* XAS spectra recorded approximately every 10 min. There were drastic changes taking place in the sample at the very beginning when the potential was altered. Every spectrum was measured twice after the potential was kept constant for 30 min. XANES and derivative of XANES spectra under the altered potential were plotted in Fig. 3a and Supplementary Fig. 13. The oxidation state of Cu was estimated by comparing the energy position of the absorption edge. It could be clearly seen that XANES dramatically changed with the decreased applied potential indicating that this was a potential-dependent process. The gradual shift of the absorption edge to low energy side revealed a fall of the Cu valence state. An obvious critical potential at −0.8 V vs. RHE could be identified. The valence state of copper remained between +1.0 and +2.0 above this potential. Notably, there was a crude transfer to +1.0 at this critical potential. Below −0.8 V vs. RHE, Cu ions gradually get more deoxidized. Especially, the position of absorption edge was in the middle of Cu foil and Cu_2_O when the potential was below −1.1 V, illustrating that the copper ions were nearly in averaged valence state of about +0.5. The changes of the atomic local structure around copper ions during CO_2_RR were captured by FT-EXAFS, as shown in Fig. 3b. Wavelet transforms for the *k*^3^-weighted Cu *K*-edge EXAFS signals at OC, −1.1 and −1.4 V vs. RHE were plot in Fig. 3c. Under open-circuit condition, FT-EXAFS data showed only one peak located at ~1.5 Å which is the typical scattering feature of Cu–N/O coordination. The FT EXAFS fitting results in Supplementary Figs. 5,  6 and Table 1 showed that CN of O/N atoms to Cu atoms was about 3.4 with an average bond length of 1.93 Å, without the Cu–Cu bond contribution. The Cu–O/N bond intensity slight decreased at −0.6 V vs. RHE. No C_2+_ products are formed at this stage in Fig. 2c. Once applying the potential of −0.8 V vs. RHE, the intensity of the scattering peak extremely descended with an additional peak at ~2.4 Å. This is a typical scattering feature of the Cu–Cu bond. The conversion of CO_2_-to-ethanol reaction occurred concurrently. The intensity of Cu–O/N scattering peak further descended with the increasing of the Cu–Cu bond as the potential decreased. Moreover, the Cu–O/N peak intensity stabilized and the Cu–Cu bond slowly increased with the potential further decreasing to −1.1 V vs. RHE. The FT EXAFS fitting results (Fig. 3d, Supplementary Figs. 5, 6 and Table 1) indicated that the coordination number of the first Cu–O/N shell gradually decreased from 3.4 to 2.0 along with decreasing potential from OC to −1.4 V vs. RHE. In addition, the average Cu–O/N bond length slightly decreased. The coordination number of the Cu–Cu bond increased to 2.7 with a bond length of 2.53 Å, slightly shorter than that in Cu foil. The FT-EXAFS analyses are in agreement with the XANES data. Note that at −1.1 V vs. RHE with the maximum FE of ethanol, the metallic Cu–Cu coordination number was 2.0 and the CN of the Cu–O/N dropped down to 2.3 concurrently.

The transformation from CuO_x_ to Cu^0^ studied by *operando* XAS during electrochemical CO_2_RR have been widely reported^30–32^. However, Cu–Cu coordination number, extracted from EXAFS data fitting was usually larger than 8 with the disappearance of Cu–O bond, indicating that Cu nanoparticles formed with average sizes more than 1nm^33^. In this work, *operando* XANES spectra revealed that Cu/N_0.14_C would not transfer to Cu^0^ totally and FT-EXAFS spectra showed Cu–N/O bond becoming steady with CN of 2.0. The stability of Cu–N coordination has been proved during electrochemical CO_2_RR^34^. These results suggested CuO clusters have almost reduced, leaving only Cu–N bond of the CuN_x_. XAFS is the technique measuring average values over all Cu species. Therefore, the fitting coordination number of Cu–N (CN = 2.0 at −1.4 V vs. RHE) was lower than the coordination number of the true active sites since a large part of Cu atoms bond to another Cu atoms (Cu–Cu CN > 2). On the other hand, considering that a definite Cu–N coordination-unsaturated structure (CN < 4) existed in Cu/N_0.14_C, we suggested that Cu–N CN of the remaining CuN_x_ is 3, x = 3. Moreover, EXAFS fitting results also indicated the Cu coordination shell switched from Cu–O to Cu–Cu with an unusual low coordination number of 2.0 at −1.1 V vs. RHE, with the maximum FE of ethanol. There are two possible structures of the in situ generated Cu species of Cu/N_0.14_C, according with the Cu–Cu CN of 2.0. One is that the formation of ultrasmall Cu moiety, Cu_n_, where *n*  =  3^35^, while the other one is a mixture of CuN_3_ sites and Cu metal particles.

We further integrated two evidence to prove the formation of the Cu_3_ moiety rather than metal particles. Firstly, we found that the potential-dependent transformation was reversible and in situ generated Cu species were highly unstable and could be easily oxidized in the absence of the applied potential. As shown in Fig. 3e and Supplementary Fig. 14, an *operando* XAS was performed on this sample after the applied potential was switched off and other conditions remained unchanged. It could be obviously seen that Cu–O/N bond increased, with Cu–Cu bond disappeared concurrently, indicating that the coordination environment reversed from Cu–Cu shell to Cu–O/N shell. Notably, the Cu–O/N bond was still lower than that of the initial fully activated sample. At last, the KHCO_3_ solution was poured out and the catalyst was in air atmosphere, and ex-situ XAS was performed on this sample. Its spectral shape was identical to that of the initial fully activated sample, as shown in Supplementary Fig. 15, suggesting the Cu species remained to be oxidized and dispersed in the air. This result agreed well with HAADF–STEM results after CO_2_RR electrolysis in Supplementary Fig. 11. The conversion process of the Cu/N_0.14_C catalyst was shown in Fig. 3f. On the other hand, we preformed *operando* XAS on the Cu/C in Supplementary Fig. 16. The XANES and FT-EXAFS of Cu/C were mostly unchanged, verifying the Cu metal particles were very stable and could not be easily oxidized. Therefore, we could rule out the existence of Cu metal particles possibility.

**Fig. 4:**
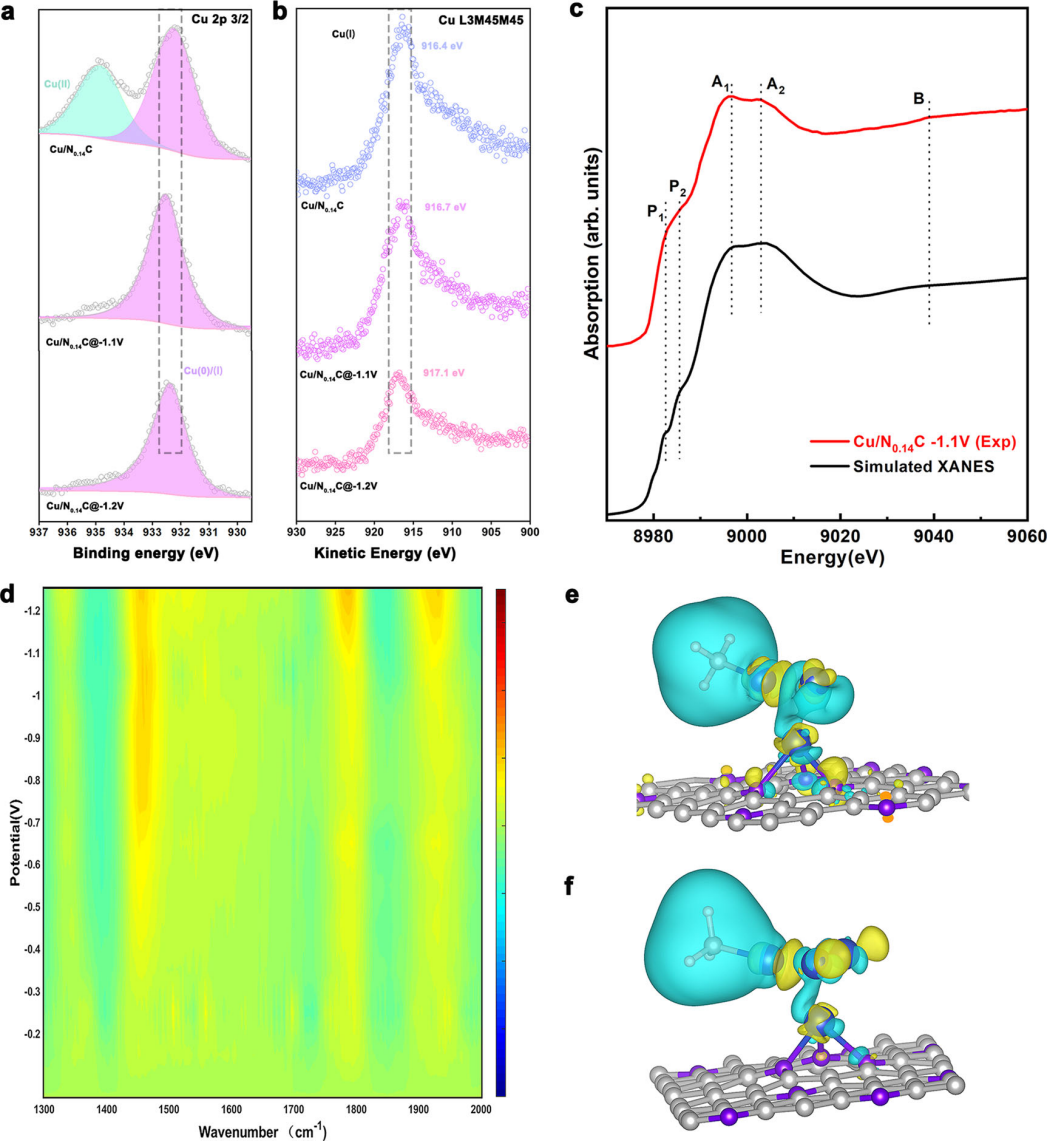
Complementary *Operando* spectra characterizations, XANES simulation and DFT calculations. **a** Quasi-in-situ XPS of the Cu 2p 3/2 for Cu/N_0.14_C. **b** Quasi-in-situ AES of the Cu L3M45M45 for Cu/N_0.14_C. **c** Comparison between the Cu K-edge XANES experimental spectra (red line) and the theoretical spectra (black line) calculated with the Cu_2_-CuN_3_ cluster. **d** *Operando* SR-FTIR of Cu/N_0.14_C. Electron density difference plot of the **e** Cu_2_–CuN_3_ cluster, **f** Cu_3_–CuN_3_ cluster. Blue and yellow represented the electron accumulation and deletion, respectively. The balls in gray, purple, blue and white represented C, N, Cu, and H atoms, respectively. All potentials were normalized to RHE.

Secondly, we carried out Quasi-in situ x-ray photoelectron spectroscopy (XPS) and Auger electron spectroscopy (AES) analysis to prove the valence of Cu species of Cu/N_0.14_C remain at +1 without Cu^0^ under the altered potential. The Quasi-in situ XPS and AES results were shown in Fig. 4a, b. The Cu/N_0.14_C showed two peaks at 934.6 eV and 932.2 eV, which corresponded to Cu^2+^ and Cu^+^/Cu^[0 26,27^. The peak at 934.6 eV disappeared at the −1.1 V vs. RHE, which indicated the Cu^2+^ was reduced during the CO_2_RR process. The peaks around 932 eV still remained. To verify the valence of the Cu, AES was employed to distinguish Cu^+^/Cu^0^ species. The peaks around 916.5 eV showed a little shift indicating that the sample would retain the valence at +1 during the CO_2_RR process^36,37^. The XPS detected the valence state information with depth sensitivity of 1–10 nm^38^. Therefore, there are no Cu metal particles existing under the electrochemical conditions. Notably, the Cu AES shifted to the higher kinetic energy with the potential reduced, indicating fewer electrons on average Cu were transferred to the nitrogen-doped carbon support of the −1.2 V vs. RHE. The catalysis electron configuration would obviously affect the adsorption of the non-polar molecule, which played an important role during the reduction process^39,40^. Probably, the ethanol productivity declined at −1.2 V vs. RHE, because the larger cluster could lead to the Cu^0^ electron configuration increasing^32,41^. Interestingly, the Quasi-in situ XPS of the Cu/C showed different changes during the CO_2_RR process, as shown in Supplementary Fig. 17. The binding energy of the Cu 2p_3/2_ peak for the pristine Cu/C was at 932.9 eV and the kinetic energy L3M45M45 peaks located at 916.4 eV correspond to the Cu^+^ species^37,42^, which may be due to the surface oxidation during exposing to air. The XPS altered completely after CO_2_RR process at −1.1 V vs. RHE. The binding energy of Cu 2p_3/2_ peak located at 932.7 eV and the kinetic energy L3M45M45 peak located at 918.5 eV corresponding to Cu^0^ ^36,43^, indicating that catalysts would not be oxidized in the transfer process. Therefore, the Cu/C without N-doping had a Cu^0^ electron configuration with high H_2_ productivity and less multi-carbon productivity. These data agreed well with the XAFS results, identifying a strong relationship between the structure of the catalysis and selectivity of multi-carbon products formation.

Hence, we can consequently conclude that the Cu–Cu bond of the *operando* spectroscopy was from in-situ generated ultrasmall Cu moieties, Cu_n_, under the electrochemical conditions, where n = 3 at −1.1 V vs. RHE. Three Cu atoms chemically bonded to each other with the Cu–Cu coordination number of 2 and one Cu atom bonded to three graphite-like N, forming the Cu_2_–CuN_3_ moiety, as shown in Fig. 3f. Such Cu_3_ moieties which are similar to copper clusters^44^ have geometric shapes of equilateral triangles, and anchored on the N-doped carbon nanosheets support. The Cu–N bonds play an important role to disperse and anchored the Cu_3_ moiety uniformly. Moreover, the coordination number of Cu–Cu increased to 2.6 with the potential decreased to −1.2 V vs. RHE, suggesting the Cu_4_ appeared with tetrahedron configuration of the Cu_3_-CuN_3_ moiety and the ethanol productivity declined accordingly. Notably, the in-situ generated Cu_n_–CuN_3_ cluster can’t exist without the applied potential. Furthermore, one CH_3_* was adsorbed to one Cu atom according to *operando* FTIRS results in the next discussion. Therefore, there are the other two oxygen atoms which were adsorbed to another Cu atom, because of the average Cu–N/O coordination number ~2. The cluster was shown in Supplementary Fig. 18. The theoretical XANES spectrum was also calculated to confirm that it was the active site based on convolution results for every considered model around each Cu atom. It could be found that the simulated result for this model could reproduce the main features of the experimental XANES at −1.1 V vs. RHE well in Fig. 4c. Two oxygen atoms may be from the adsorbed groups, such as OH*, OCHO*(or CO_2_), as shown in Supplementary Fig. 19a, c. The experimental spectra were both properly reproduced in Supplementary Fig. 19b, d. The simulated spectra were similar to the spectrum that was calculated without the adsorbed groups. A slight change in the structure of the 2nd shell does not significantly affect the simulated XAFS spectrum, especially for the H atom. On the other hand, the XANES spectra of the references with different local structures were used to compare with XANES of the Cu/N_0.14_C at −1.1 V vs. RHE and rule out Cu–N_2_ site, Cu–N_3_ site with trigonal planar geometry, Cu–N_4_ sites (including tetrahedral geometry, square planar geometry and Cu–N_3_ with CH_3_*) and different sizes Cu nanoparticles as shown in Supplementary Figs. 20–23. We also tried linear combination fitting (LCF) of XANES of Cu NPs with different sizes and various Cu–N_4_ sites, as shown in Supplementary Fig. 24. We could not obtain a fitting spectrum which was matched with *operando* XANES at −1.1 V vs. RHE. The main features of LCF result were notable different from that of *operando* XANES. Therefore, the possibility of combining for Cu NPs with different sizes and various Cu–N_4_ sites could be excluded.

In order to verify that Cu_2_–CuN_3_ clusters in-situ generated are truly metastable catalyst and the potential-dependent reversibility was real chemical reaction, the freeze-quench approach was used to freeze the redox state and local structure of the catalyst and measured XAFS data at liquid helium temperatures (18 K), as shown in Supplementary Fig. 25a. The X-ray edge energy as well as the spectral shape of the freeze-quench and *operando* spectra do not differ significantly in Supplementary Fig. 25b–d. This comparison verified that the freeze-quench and *operando* method access the same electrochemical state of the Cu/N_0.14_C (same oxidation state and local structure). The spectra which were measured for three times could be perfectly overlapped in Supplementary Fig. 25e–g. These results indicated that Cu_2_–CuN_3_ clusters oxidized without the applied potential was the chemical reaction and Cu_2_–CuN_3_ clusters in-situ generated material were truly metastable catalysts.

Overall, *Operando* XAS study revealed that a transformation from Cu/N_0.14_C to unique ultrasmall Cu_n_ moiety and presented the structure-activity relationships of this catalyst, indicating the in-situ generated Cu_2_–CuN_3_ cluster is the optimal site for CO_2_ reduction to ethanol.

To further reveal the key adsorbed intermediates in the catalytic reaction, we carried out Synchrotron radiation *operando* Fourier-transform infrared spectroscopy (SR-FTIRS). No obvious absorption band was observed on scanning the applied potential ranging from −0.1 V to −0.7 V vs. RHE (Fig. 4d). Interestingly, distinct vibration features were observed at 1450 cm^−1^ when applied potentials were lower than −0.7 V vs. RHE, which could be assigned to the antisymmetric methyl groups vibration of CH_3_* ^45,46^, an important intermediate for C_2_ formation rates. Furthermore, when the applied potential was below −1.1 V vs. RHE, two new bands at ~ 1780 cm^−1^ and 1920 cm^−1^ could be ascribed to the surface-bound C=O species^12,47^ and electrogenerated CO bound to copper surface^12,48^ suggesting H* radicals were consumed by other products at −1.2 V vs. RHE, which was consistent with the electrochemical results (Fig. 2c). The FE of the ethanol reached a high point at −1.1 V vs. RHE and then decreased obviously at lower potential to accompany the hydrogen rate increased. The FE of ethanol reversed at −1.2 V vs. RHE may be due to the Cu cluster enlarging that lead to the competing reaction of HER and CO_2_RR hydrogenation to CH_4_ enhancing, which blocked the CO_2_ to ethanol^41,49^. These FTIRS analysis showed uniformity with *operando* XAS and XPS results. On the other hand, the infrared bands for CH_3_* over the Cu/C catalyst were not observed even after applying potential at −1.2 V vs. RHE in Supplementary Fig. 26. The intensities were much lower than those over the Cu/N_0.14_C. These results were consistent with the low C_2+_ formation rates of the Cu/C catalyst, further confirming the unique structure of Cu/N_0.14_C under the electrochemical condition. Therefore, FTIR results provided experimental evidence for the conversion of CO_2_-to-ethanol reaction mechanism and demonstrated that CH_3_^*^ was the major intermediates covered the catalysis. To further investigate the property of the adsorption species, the isotope labelling *operando* FTIR was measured under the reaction condition, as shown in Supplementary Fig. 27. With H_2_O replaced by D_2_O in the electrolyte, the peak shifted to 1190 cm^−1^ obviously indicating that it could be assigned to the vibration of hydrogen group (CD_3_*). This result was consistent with previous speculate. Therefore, the FTIR results suggested that the adsorption of CO_2_ was the rate-determining step after CH_3_^*^ formed. It may be highly affected by mass transfer process. The results are consistent with previous reports whereby the selectivity of C_2+_ products formation increased with the density of CO_2_^50,51^.

### Density functional theory calculations

Theoretical investigations were conducted based on DFT calculations to further understand the reaction mechanism of the Cu_2_–CuN_3_ cluster with CH_3_^*^ adsorbed. A supported Cu_2_–CuN_3_ cluster model with a Cu bonding with three N atoms on N-doped carbon nanosheets was proposed to be the structure of active sites with an applied potential of −1.1 V vs. RHE in Supplementary Fig. 28a. For comparison, we built a model of Cu_3_–CuN_3_ cluster in Supplementary Fig. 28b that represent for the growth of Cu clusters and may be the active site for the more negative applied potential. The calculated average partial charge of three copper atoms in the Cu_2_-CuN_3_ cluster was +0.20 e^−^ because of the charge transfer between the cluster and N-doped carbon nanosheets. The charge exchange primarily occurred with the Cu atom bonding with N atoms. The other two Cu atoms changed slightly before the reaction occurred, which profited from the absorption of C atoms. This was similar to the Cu_3_–CuN_3_ cluster. The difference was that the average partial charge was +0.15 e^−^. Interestingly, the charge distribution between Cu atoms was charge-asymmetry as shown in Fig. 4e f. The major charge transfer occurred at Cu–N_3_ site. The N-doping played an important role to adjust the electron transfer and further charge distribution between the Cu atoms and N-doped carbon interface.

**Fig. 5:**
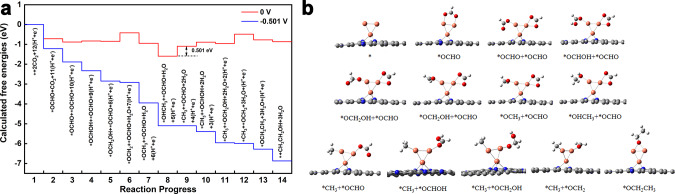
DFT calculations of CO_2_RR activity. **a** Reaction progress of CO_2_ to CH_3_CH_2_OH on the Cu_2_–CuN_3_ at 0 V and −0.501 V applied potential. The * showed the reaction site. The state with the highest energy barrier (0.501 eV) is 8 → 9. All states involving the transfer of the (H^+^ + e^−^) pair were electrochemical reaction states, except for the 12 → 13 state (CH_3_* + OCH_2_* → OCH_2_CH_3_*), which was the C–C bond formation and not influenced by applied potential. **b** Local structures of the active site and intermediate state. The balls in brown, blue, red, gray and white represent Cu, N, O, C, and H atoms, respectively.

Theoretical investigations were conducted based on DFT calculations to further understand the reaction mechanism of the Cu–N_3_ with triangle pyramid geometry, Cu–CuN_3_, Cu_2_–CuN_3_, and Cu_3_–CuN_3_ in Supplementary Fig. 20c, f, g, h. The supported CuN_3_, Cu–CuN_3_, Cu_2_–CuN_3_ and Cu_3_–CuN_3_ models with a Cu bonding with three N atoms on N-doped carbon nanosheets which were proposed to be the structure of active sites with the applied potential and represents the growth of Cu clusters that may be the active site for the formation of ethanol according the results of *operando* XAS analysis. The reaction pathway of CO_2_RR to CH_3_CH_2_OH for the Cu_2_–CuN_3_ cluster was investigated combined by the computational hydrogen electrode approach as shown in Fig. 5a, b, Supplementary Fig. 28, and Supplementary Data 1–13^52^. The *OCHO mechanism was preferred for our catalysts than the *CO mechanisms, due to the production of CO was suppressed along with the formation of ethanol at the potentials below −0.8 V as shown in Fig. 2b. CO_2_ molecules interact with two exposed Cu atoms, respectively, forming coadsorbed (*OCHO + *OCHO) (State 3). One adsorbed *OCHO was firstly reduced to form *CH_3_ + *OCHO (State 9), and then the other *OCHO was reduced to construct *CH_3_ + *OCH_2_ (State 12), that went through the C–C bond formation to CH_3_CH_2_OH. Similar reaction processes based on *OCHO mechanism were considered for Cu_3_–CuN_3_, as well as CuN_3_ and Cu–CuN_3_ which contained only one active Cu site, as shown in Supplementary Fig. 29. The calculated overpotential (−0.501 V) for Cu_2_–CuN_3_ cluster was much lower than that of CuN_3_ (−1.685 V), Cu–CuN_3_ (−1.368 V) and Cu_3_–CuN_3_ (−0.699 V), implied that the Cu_2_–CuN_3_ observed by *operando* XAS is the real active site for reduction of CO_2_ to ethanol, as shown in Supplementary Fig. 30. Furthermore, there were relatively high energy barriers for State 8 → 9 (−0.501 eV) and State 11 → 12 (−0.467 eV) in the reaction process of Cu_2_–CuN_3_, both of which containing –CH_3_ and easy to be detected in accordance with our FTIR results.

The hydrogen evolution reaction is a competing reaction with CO_2_RR^53,54^. Therefore, the competing relationship between CO_2_RR and HER should be considered. The adsorption energy was calculated to further investigate the difference between Cu_2_–CuN_3_ and Cu_3_–CuN_3_. The adsorption energy of H* showed that the Cu_3_–CuN_3_ model (−0.36 eV) had a much lower energy than the Cu_2_–CuN_3_ model (+0.15 eV). The CO_2_ adsorption energy for Cu_2_–CuN_3_ and Cu_3_–CuN_3_ has been calculated and the result shown that the adsorption energy of CO_2_ for Cu_2_–CuN_3_ and Cu_3_–CuN_3_ are +0.24 eV and +0.18 eV, respectively, implying the adsorption capacities for Cu_2_–CuN_3_ and Cu_3_–CuN_3_ are close. However, the corresponding adsorption energy is +0.15 eV (Cu_2_–CuN_3_) and −0.36 eV (Cu_3_–CuN_3_), respectively, indicating the adsorption capacities of H for Cu_3_–CuN_3_ are much stronger than that for Cu_2_–CuN_3_. These results shown the adsorption processes of both CO_2_ and H at the Cu_2_–CuN_3_ are non-spontaneous processes. On the contrary, for Cu_3_–CuN_3_, the adsorption of the H is spontaneous process, while the adsorption of CO_2_ is still non-spontaneous process, illustrating the H adsorption is advantage state for the competing reaction. Therefore, the larger amounts of H were adsorbed on the Cu_3_–CuN_3_ sites, which preempted the adsorption sites of CO_2_ and reduced the amount of ethanol produced. It corresponded with the competing reaction of H_2_ and CH_4_ increasing when the potential decreased.

CH_3_^*^ was the key intermediate for C_2+_ formation rates and it occurred when the potential decreased to −0.6 V vs. RHE. This result suggested the rate-determining steps (RDS) altered when the potential at decreased to −1.2 V vs. RHE, combining the FTIR results in Fig. 4d. To further analysis why RDS changed during the reaction, the different charge density for Cu_2_-CuN_3_ (with/without CH_3_^*^) and Cu_3_-CuN_3_ (with/without CH_3_^*^) was calculated as showing in the Fig. 4e, f and Supplementary Fig. 31. The charge density differential diagram indicated the nonbonding Cu atoms in the Cu_2_–CuN_3_–CH_3_ got 0.15 e^−^ and the charge accumulation occurred. These results showed that charge-asymmetry effected intensified by CH_3_* adsorption. Due to the charge accumulation, a polarization electrical field was presented that could modulate the CO_2_ reduction reaction and reduce the thermodynamic energy barrier for the reaction^55^. The electron-rich Cu atoms enhanced the boundary oxygen sites of the adsorbed CO_2_, which protected the oxygen end from being protonated and occupied the valence electron of carbon to avoid the C=C double bond formation^56^.

### Conclusions

In summary, we systematically investigated a catalyst in which CuO clusters are supported on nitrogen-doped carbon nanosheets (Cu/N_0.14_C) for efficient CO_2_ electroreduction. The catalysts delivered high C_2+_ products FE (~73%), which included an ethanol FE of 51% at the potential of −1.1 V vs. RHE and the current density of −14.4 mA/cm^−2^ in 0.1 M KHCO_3_ electrolyte. *Operando* XAS and XPS results revealed a potential-dependent structure transformation from CuO cluster to Cu_n_–CuN_3_ moiety, which is a reversible process. Once Cu_2_–CuN_3_ clusters have been formed at −1.1 V, CO_2_ conversion to ethanol occurs with the maximum FE, indicating the Cu_2_-CuN_3_ cluster is the optimal site. The N-doping played an important role to disperse the reduced Cu_n_ cluster uniformly and adjust charge distribution between metal atoms and substrate. *Operando* FTIR and theoretic calculations results demonstrated that CH_3_^*^ was the major intermediates and Cu_2_–CuN_3_ clusters were charge-asymmetric sites that are intensified by CH_3_* adsorbing. The charge-asymmetric sites are responsible for the outstanding asymmetry ethanol formation. Overall, our findings suggest a strategy engineering the pre-catalysts to modulate the electron transfer and in-situ generate ultrasmall metal clusters anchored on the substrate as high dispersion asymmetric sites, which is the key factor to facilitate the formation of asymmetric products. It would extend to the single-atomic site, oxide composite structure, and metal-support effect. These catalysts have great potential to exhibit novel catalytic properties such as more efficient catalytic performance and higher selectivity compared to the single atom catalysts and oxide catalysts.

## Methods

### Preparation of Cu/N_x_C and the comparison samples

Typically, 100 mg copper phthalocyanine and 1.0 g dicyandiamide were continuously mixed in ethanol and entirely dried. Subsequently, the dried mixture was pyrolyzed for 2 h in the N_2_ atmosphere at different temperature to obtain a series of Cu/N_x_C samples with different nitrogen content (x = 0.14, 0.11, 0.02, corresponding pyrolysis temperature at 800 °C, 900 °C, 1000 °C, respectively. x is mass content ratio of nitrogen to carbon). Collect obtained black powder after pyrolysis and directly used for further characterizations and catalytic performance test. Cu/C was prepared with copper chloride and trimesic acid. The pyrolysis temperature was 800 °C and other synthesis conditions were the same as above.

### Characterization

TEM images were carried out by the JEOL JEM-2100F microscope (200 kV). AFM was obtained on SPM-960 AFM. Typically, small amount of sample was ultrasonically dispersed in the aqueous solution. For AFM measurement, 10 μL of the completely dispersed solution was carefully dropped on a flat mica sheet, and then placed it in a fume hood to air dry. For TEM, 10 μL of the dispersed solution prepared above was dropped on a copper mesh with carbon support film. EDS elemental mapping and HAADF-STEM were characterized by a JEOL ARM-200 microscope (200 kV) with a probe spherical aberration corrector. X-ray powder diffraction (XRD) spectra were observed by RigakuTTR-III XRD of Cu *Kα* radiation (λ = 1.5418 Å). XPS spectra were acquired on a PerkinElmer Physics PHI 5300 spectrometer^57^.

### Electrochemical measurements

Electrochemical measurements were accomplished in a gas-tight H-type cell at CHI 760E electrochemical workstation (Shanghai Chenhua, China). For preparation of the working electrode, 2 mg of the catalyst was dissolved in 500 μl solution (including 10 μl Nafion solution (5 wt %) and deionized water/ethanol (volume ratio, 1:4)) to form a well-dispersed catalyst ink. Furthermore, we dropped 10ul ink on the glassy carbon electrode. The catalyst loading is about 0.2 mg/cm^2^. The Ag/AgCl electrode and platinum plate were employed as the reference electrode and counter, respectively. The cathodic chamber and anodic chamber were separated by Nafion 117 membrane. All the potential was reported versus RHE. The conversion between different electrode potentials used the following formula: E_RHE_ = E_Ag/AgCl_ + 0.197 V + 0.0591 × pH. LSV (the scan rate of 10 mV·s^−1^) and CV experiments (50 mV·s^−1^) were implemented to test the catalytic performance in CO_2_-saturated 0.1 M KHCO_3_ electrolyte, respectively^57^. Comparison of CO_2_RR activities for the other catalysts were shown in Supplementary Table 3.

### *Operando* XAFS Measurements

The computer-controlled electrochemical analyzer was used to conduct electrochemical measurements. The catalyst modified carbon paper, Pt plate and Ag/AgCl (KCl-saturated) electrode were employed as the working electrode, counter electrode and reference electrode, respectively. The plexiglass electrochemical cell which had the flat wall with a circular hole of 15 mm diameter. was applied for *operando* XAFS measurements. Cu/N_x_C catalyst coated carbon paper contacted with the copper slip and the catalyst layer faced inward. Afterwards we poured KHCO_3_ solution into the cell which was connected to the electrochemical station with the copper tape. The organic glass upper cover of the cell with the Ag/AgCl reference electrode was used to fix the distance between reference electrode and working electrode from start to finish^57^. *Operando* XAFS data were measured with fluorescence mode using the Ar-filled Lytle detector.

### *Operando* SR-FTIR Measurements

*Operando* SR-FTIR measurements were conducted at the infrared spectroscopy and microspectroscopy station (BL01B) of National Synchrotron Radiation Laboratory (NSRL) ^58^. The CHI 660E electrochemical workstation (Shanghai Chenhua, China) was used to apply voltage to a self-made infrared reflection cell. The infrared spectrums were collected using Bruker Vertex 70 V/s FTIR spectrometer with nitrogen cooled mercury cadmium telluride detector, and the synchrotron radiation infrared beam was focused on the reflection cell using Bruker Hyperion 3000 microscope equipped with a ×15 objective. In the measurements, the gap between the working electrode and the ZnSe window was carefully maintained on the order of microns to prevent excessive water absorption. All spectrums were collected using single-potential alteration FTIR spectroscopy and were acquired by averaging 128 times scan with a 4 cm^–1^ resolution, and the background spectrums was conducted under open-circuit voltage, the voltage ranges for conducting measurements were 0 V to –1.2 V with an interval of 0.1 V.

### Quasi-in situ XPS and AES

Quasi-in situ X-ray photoelectron spectroscopy was conducted of catalysts drop-casted on carbon paper as same as mentioned in Electrodes section. Before test the electrolytes should be CO_2_-saturated to remove the dissolved oxygen in the solution. Samples were conditioned at different potential for 30 min as one conditioning step. After that the samples was moved to the XPS vacuum chamber without extra exposed to the air. That would avoid the air to influence the catalysts to keep the authentic state under the CO_2_RR process. XPS (ESCALAB 250X) was measured at Analytical Instrumentation Center, Shanghai Tech University. It was used to characterize the chemical bonding character of the obtained catalysts using a monochromatic of 1486.6 eV (Al *Kα* source). All XPS spectra were corrected by C 1*s* peak with the binding energy at 284.6 eV. All of the reagents were used without further purification.

### Freeze-quench XAS

The Cu/N_0.14_C was electrodeposited on a carbon paper electrode, which was an integral part of the XAS sample holder. The Cu/N_0.14_C was equilibrated potentiostatically for 20 min at −1.1 V vs. RHE. The Cu/N_0.14_C electrode was rapidly frozen by immersion in liquid nitrogen. The chemical reaction is extremely slow at low temperature environment and the chemical state of the catalyst was well preserved for XAFS data collection periods. X-ray absorption spectra at the Cu K-edge were collected at beamline BL14W1 of the SSRF using a Lytle detector with the Ni filter. The measurements were performed at 18 K using a cryostat (ARS). The excitation energies (scan range 8779–9779 eV) were selected by a Si (111) double-crystal monochromator.

### Computational methods

DFT calculations were performed within the frame of plane-wave-based density functional theory (DFT) by VASP package. The generalized gradient approximation (GGA) in the Perdew−Burke−Ernzerhof (PBE) formalism was adopted to treat the electron exchange and correlation energy. The cut-off energy was set to be 500 eV. For structural optimization, 3 × 3 × 2 k-points meshes with a the original Monkhorst-Pack scheme was used and the convergence of energy and force were 10^−6^ eV and 0.01 eV  Å^−1^, respectively. 6 × 6 × 4 k-points meshes were used for static calculations. A 20 Å vacuum layer in the z-axis direction was added to avoid the interfere of the layer image coupling caused by the periodic boundary conditions^59^. For more details, please see Supplementary Figs. 20,  28,  29,  31 and Supplementary Data file 1–13.

## Supplementary information

Supplementary Information

Description of Additional Supplementary Files

Supplementary Data 1

Supplementary Data 2

Supplementary Data 3

Supplementary Data 4

Supplementary Data 5

Supplementary Data 6

Supplementary Data 7

Supplementary Data 8

Supplementary Data 9

Supplementary Data 10

Supplementary Data 11

Supplementary Data 12

Supplementary Data 13

## Data Availability

The data supporting the findings of this work are available within the article and its Supplementary Information files. The DFT data for cluster and reaction pathway as cif files are provided as a Supplementary Data file 1–13. All the data reported in this work are available from the authors on reasonable request. Source data are provided with this paper.

## Source data

Source Data
