# Supplementary material for: Complementary Operando Spectroscopy identification of in-situ generated metastable charge-asymmetry Cu2-CuN3 clusters for CO2 reduction to ethanol
Source: Nat Commun. 2022 Mar 11;13:1322. doi: 10.1038/s41467-022-29035-8 (PMC8917205; doi:10.1038/s41467-022-29035-8)
Supplement: Supplementary file 1 — Supplementary Information [file 41467_2022_29035_MOESM1_ESM.pdf]

Supplementary Information for

Complementary *Operando* Spectroscopy identification of metastable charge-asymmetric Cu_2-CuN_3 clusters generated *in-situ* for CO_2 reduction to ethanol

Su *et al.*

1. Supplementary Methods

Materials. Copper phthalocyanine, dicyandiamide, $KHCO_3$, ethanol was purchased from Sinopharm Chemical. Copper chloride and trimesic acid were purchased from Alfa Aesar. Deionized water was used for all synthesis.

CO_2RR products analysis. Gas chromatography (GC, Shimadzu 2010) system was performed to detect CO_2 reduction gas products, equipped with a thermal conductivity detector (TCD) and flame ionization detector (FID). The inlet of GC was connected to the outlet of the cathode compartment for real-time monitoring. ^{1}H NMR experiments was carried out by NMR (Bruker 400 MHz) to analyze the liquid phase products. In detail, 300 μL of the catholyte after reaction was mixed with 300 μL of D_2O containing 5 mM DMSO as an internal standard. Typically, NMR was tested with 2 s pre-saturation delay and 2 s relaxation delay. Chronoamperometric measurements were performed to obtain the Faradaic efficiency (F.E.) of products.

Gaseous Products.

$$FE_{gas}(\%) = \frac{Q_{gas}}{Q_{total}} \times 100\% = \frac{\left(\frac{v}{60s/min}\right) \times \left(\frac{V_{gas}}{\frac{24000cm^3}{mol}}\right) \times N \times F \times 100\%}{j}$$

v: gas flow rate.

V_{gas}: H_2 or CO volume concentration.

N: electrons number needed to form one molecule of H_2 or CO.

F: Faraday constant as 96485 C/mol.

j: recorded current.

Liquid Products.

$$FE_{Liquid}(\%) = \frac{Q_{Liquid}}{Q_{total}} \times 100\% = \frac{n_{Liquid} \times N \times F \times 100\%}{j \times t}$$

N_{Liquid}: liquid products amount in the cathodic compartment.

t: reaction time.

XAFS measurements. N K-edge and C K-edge XAS spectra were carried out at BL12B station from the bending magnet in National Synchrotron Radiation Laboratory (NSRL). From 0.1 to 1 keV there are three gratings covering photon energies at BL12B. The energy resolution is ~0.2 eV. Catalysts were coated carbon tape for XAS. TEY mode was used to record the XAS data.

Copper K-edge XAFS data of catalysts were measured at BL14W1 beamline in Shanghai Synchrotron Radiation Facility which is operated at 3.5 GeV with the top-up current of 200 mA. The synchrotron radiation was monochromatized using the Si (111) liquid nitrogen cooled double-crystal monochromator. The incident X-ray intensity was monitored with the N_2-filled ion chamber (I_0) in front of catalysts. XAFS data were measured with fluorescence mode using the Ar-filled Lytle detector. The X-ray energy was calibrated using the Cu metal foil for Cu K-edge. The XAFS spectra of references were carried out with the transmission mode using the N_2-filled ionization chamber (I_1) behind references.

XAFS Data Analysis. EXAFS data were got by taking out the background and normalizing with the edge-jump by Athena and Artemis in the IFEFFIT package. Cu K-edge k^3-weighted χ(k) data of k-space ranging from 2.0–11.5 Å^{-1} were FT to R-space. Quantitative curve fittings were carried out to obtain the structural parameters around copper atoms by the ARTEMIS module[1].

EXAFS equation was shown as following:

$$\chi(k)=\sum_j \frac{N_j S_o^2 F_j(k)}{k R_j^2}\exp[-2k^2\sigma_j^2]\exp[\frac{-2R_j}{\lambda(k)}]\sin[2kR_j+\phi_j(k)]$$

$F_j(k)$ (backscattering amplitude of curved-wave), $\phi_j(k)$ (phase shift) and $\lambda(k)$ (mean free path) were calculated by FEFF8.2. S_0^2 (amplitude reduction factor) was fixed in the fitting. N_j (coordination number of atoms in the j_{th} shell), R_j (distance between the absorbing copper atom and j_{th} shell atoms), σ_j (Debye-Waller factor of the j_{th} shell) and ΔE_0 (edge-energy shift) were fit freely.

XANES simulations were carried out with the FDMNES program, which is based on the Finite Difference Method (FDM) to solve the Schrödinger equation and uses the Green formalism on the muffin-tin potential.[2] The final states were calculated in this cluster with a fixed radius, with the atoms inside the sphere considered in the calculation. To achieve a balance between the good calculation and time spending, a radius of 6 Å was selected for the sphere. The models were performed for every considered Cu atom to develop corresponding Cu speciation specific. The features of simulated XANES spectrum were adjusted by modifying the convolution parameters dependent theoretical XANES systems.

2. Supplementary Figures and Tables

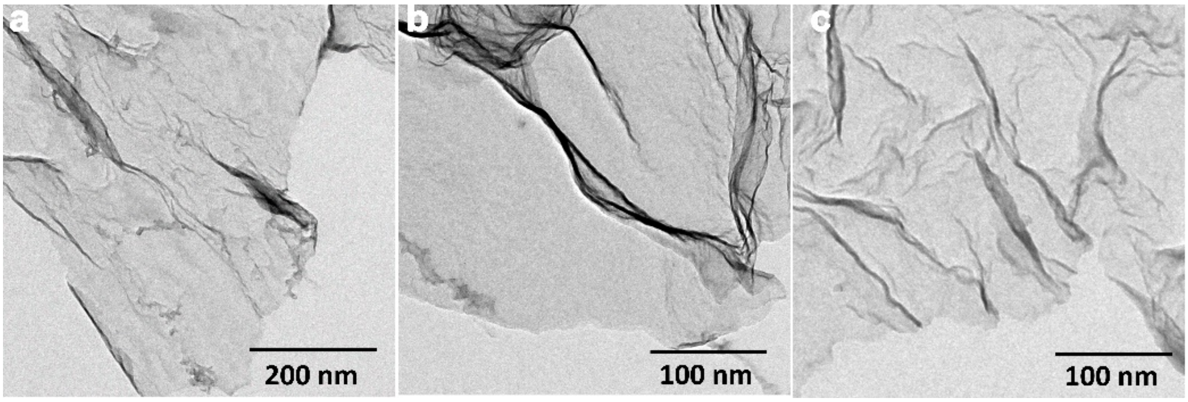

Supplementary Fig. 1 TEM characterizations. **a** NC. **b** Cu/$N_{0.02}$C. **c** Cu/$N_{0.11}$C.

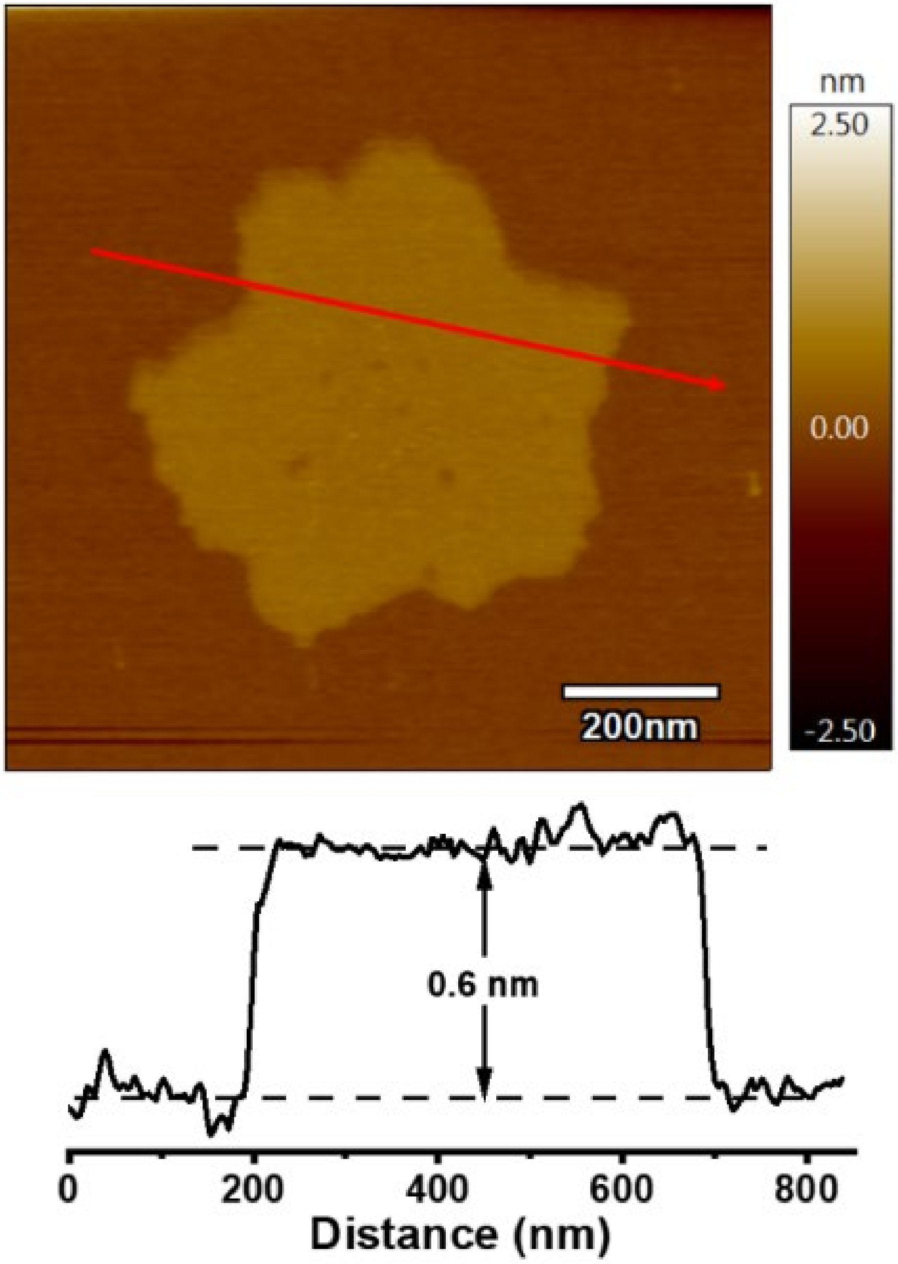

Supplementary Fig. 2 The AFM image of Cu/$N_{0.14}$C.

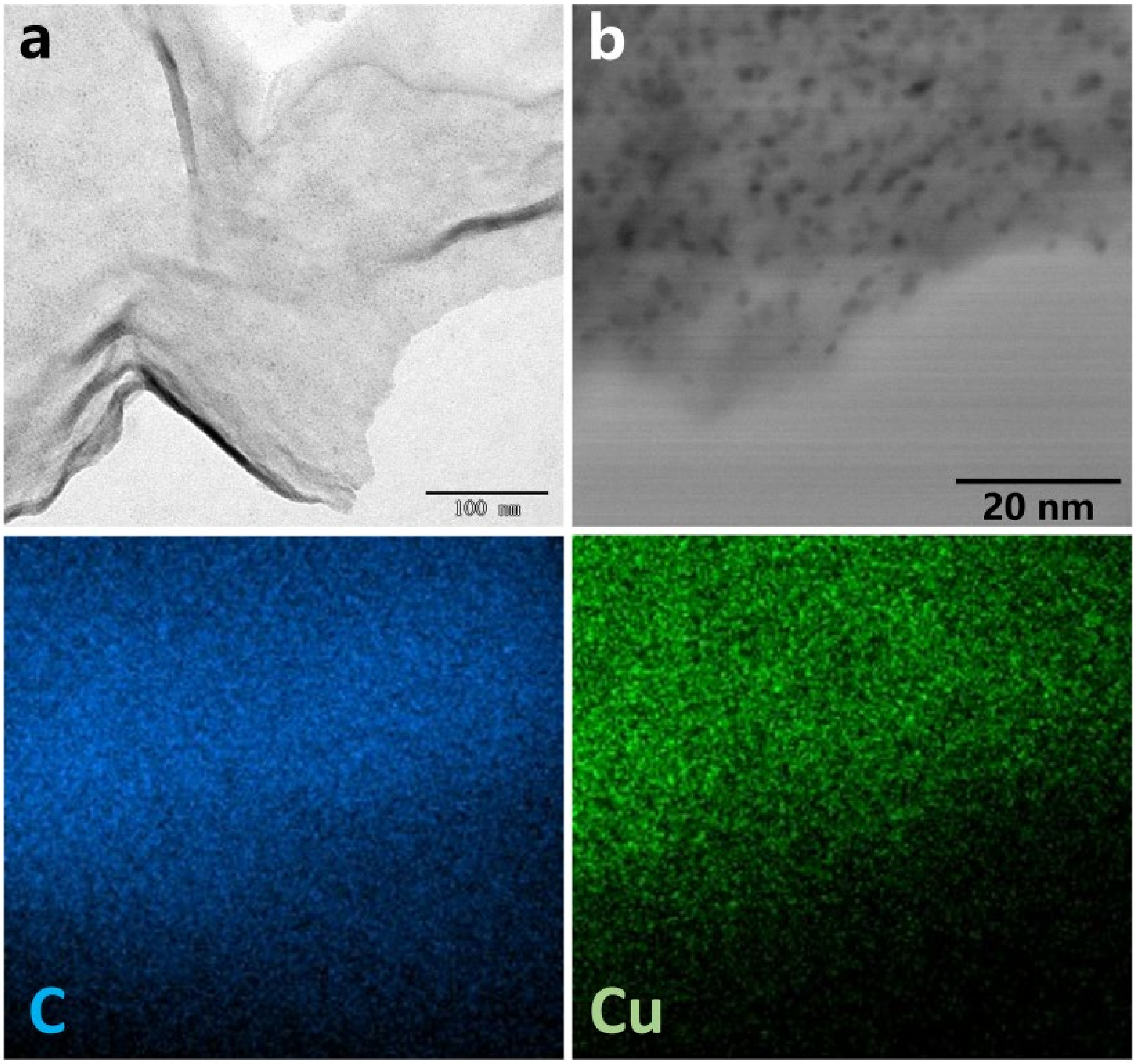

Supplementary Fig. 3 The TEM and EDS elemental mapping images of Cu/C.

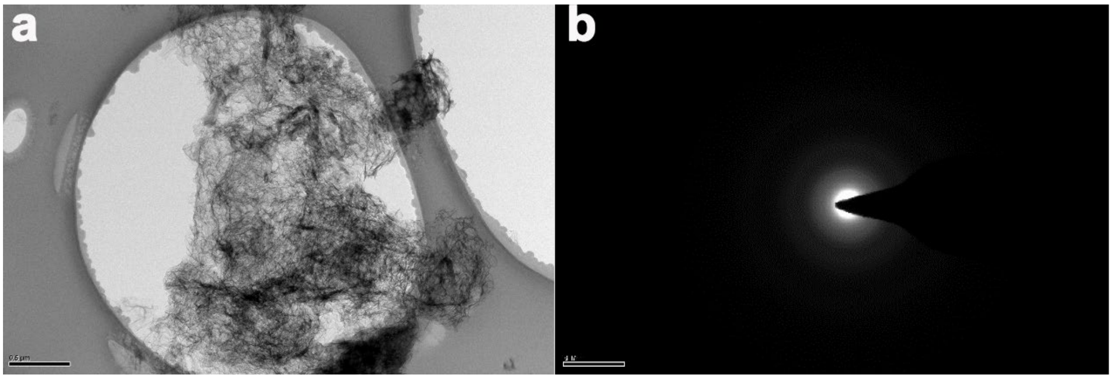

Supplementary Fig. 4 TEM characterizations. a The TEM of Cu/$N_{0.14}$C. **b** The electron diffraction pattern of Cu/$N_{0.14}$C.

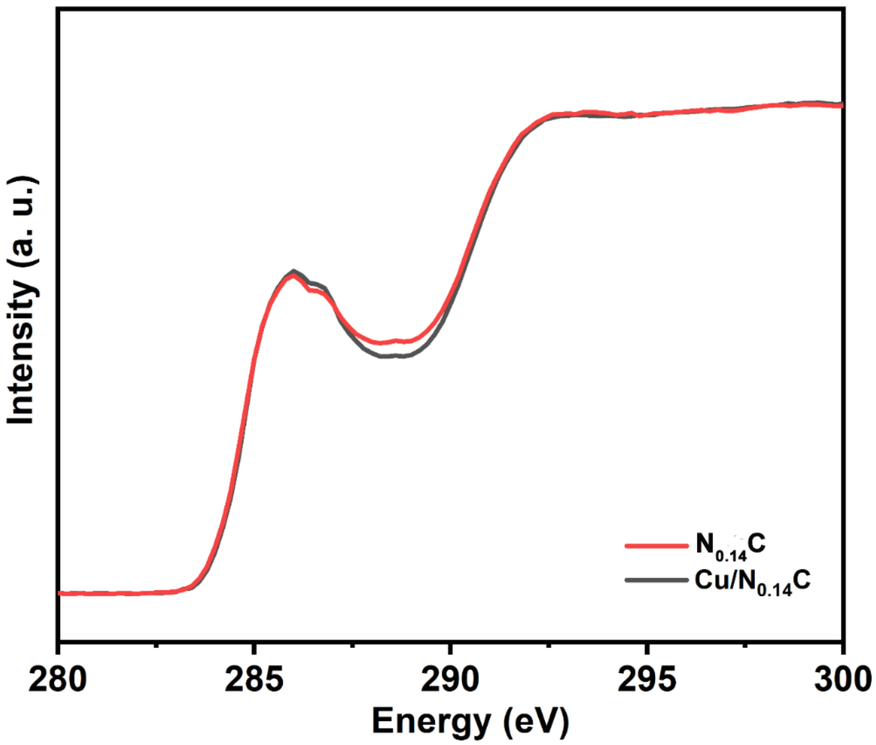

Supplementary Fig. 5 The C *K*-edge XAS spectra of Cu/$N_{0.14}$C and $N_{0.14}$C.

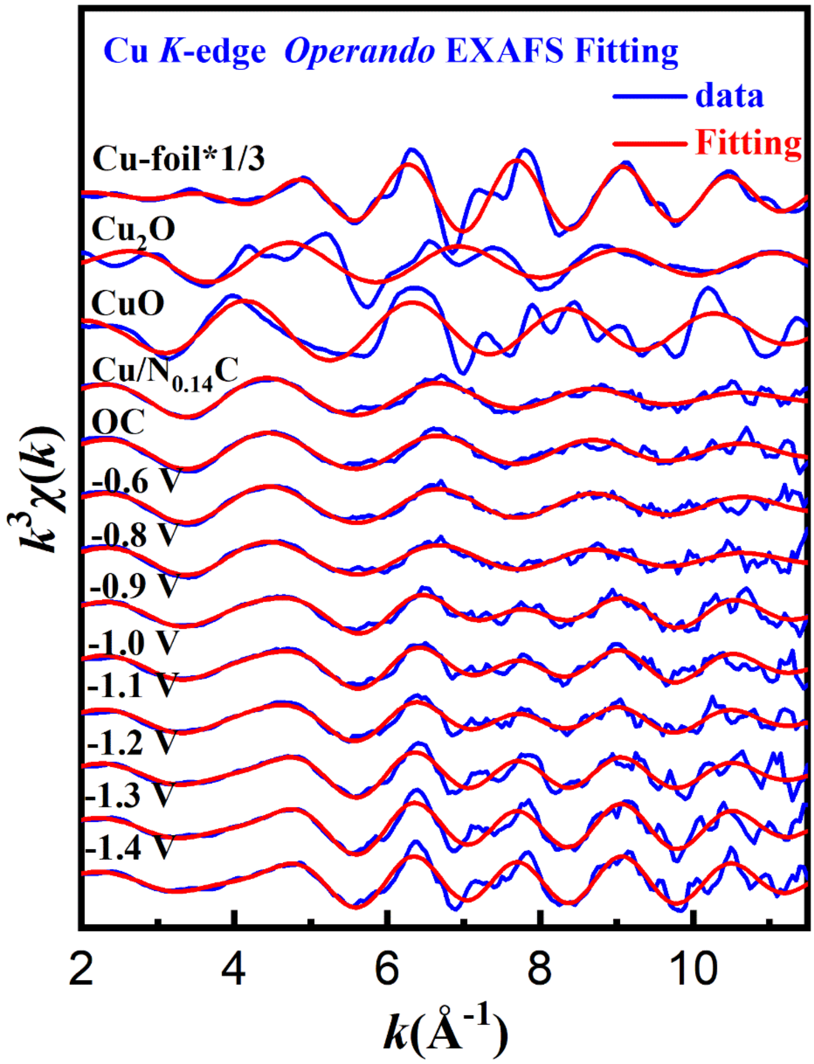

Supplementary Fig. 6 The FT-EXAFS fitting of Cu *K*-edge in *k*-space for Cu/$N_{0.14}$C;

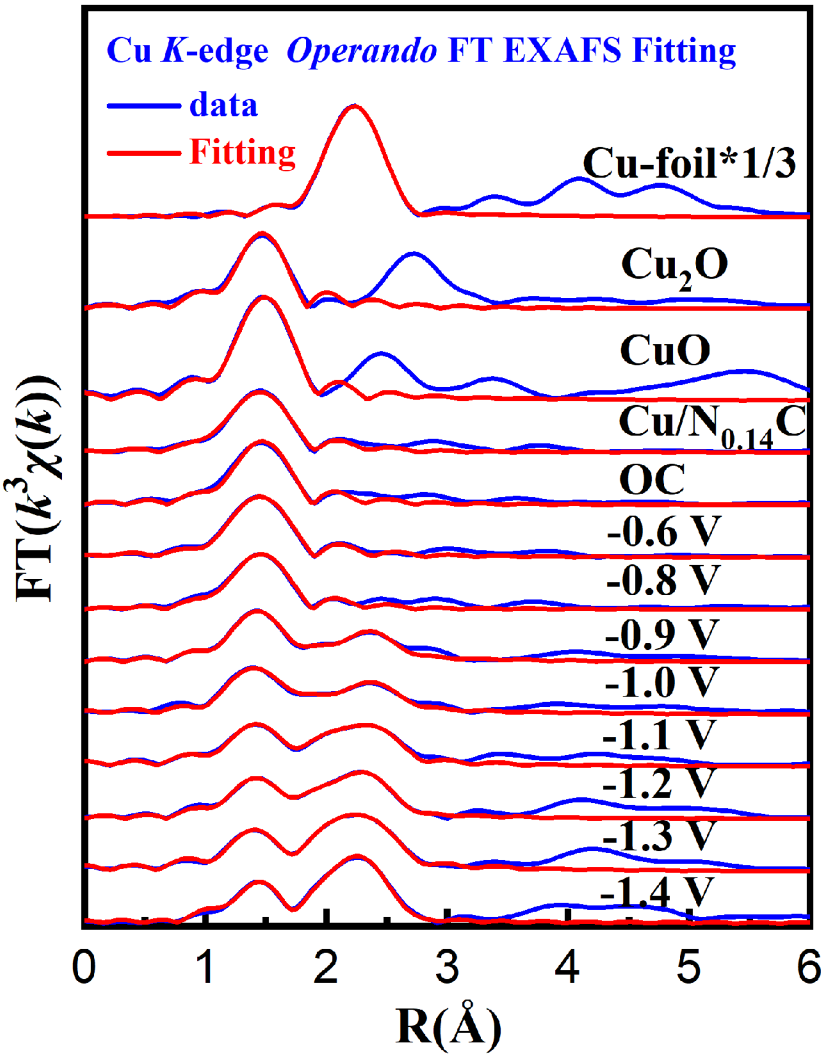

Supplementary Fig. 7 The FT-EXAFS fitting of Cu *K*-edge in R-space for Cu/$N_{0.14}$C

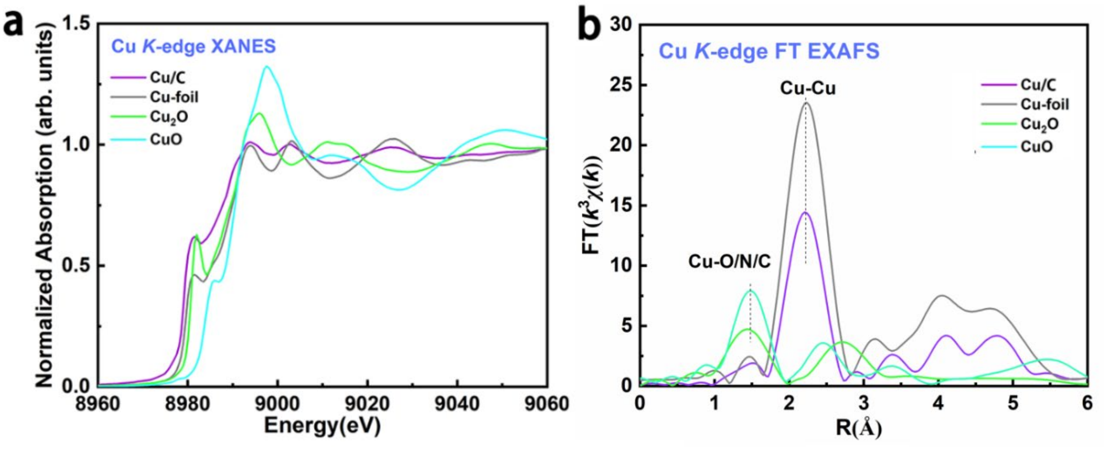

Supplementary Fig. 8 XAFS characterizations of Cu/C. a Cu K-edge XANES spectra of Cu/C with the references. **b** FT-EXAFS spectra.

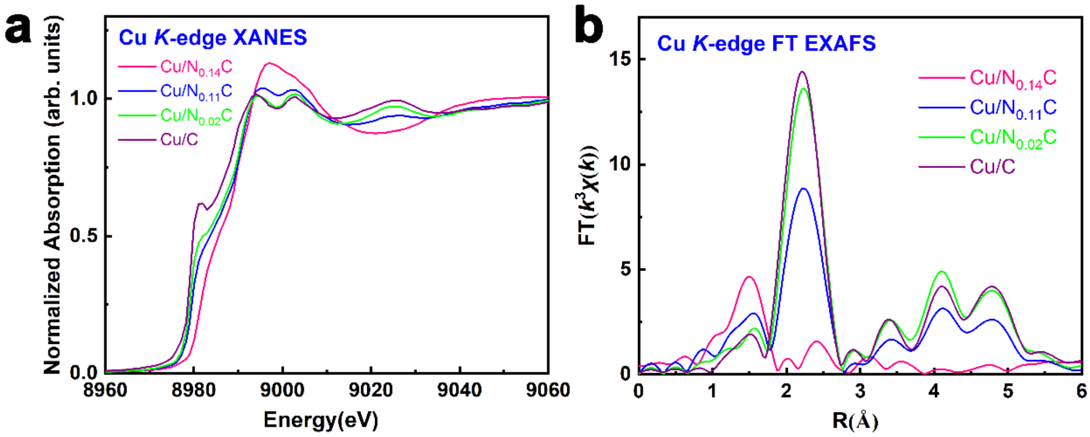

Supplementary Fig. 9 XAFS characterizations of Cu/N$_x$C. a Cu K-edge XANES spectra of Cu/N$_{0.14}$C, Cu/N$_{0.11}$C, Cu/N$_{0.02}$C as well as Cu/C. **b** FT-EXAFS spectra.

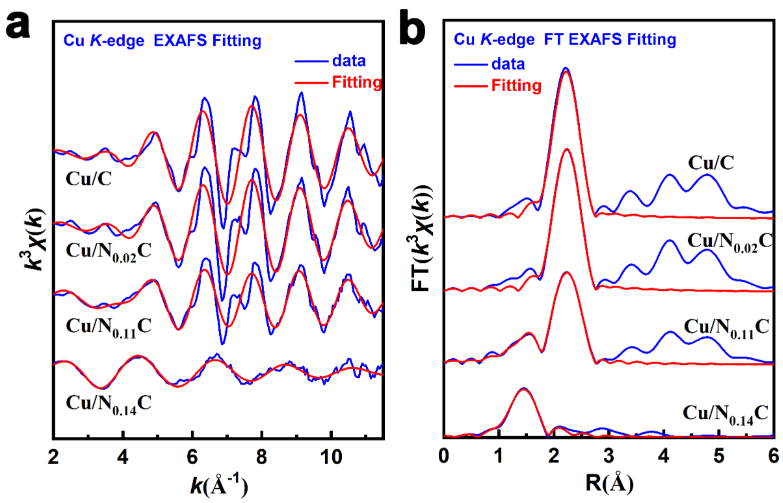

Supplementary Fig. 10 Fitting results of Cu/N_xC. a The FT-EXAFS fitting of Cu *K*-edge in R-space for Cu/$N_{0.14}$C, Cu/$N_{0.11}$C, Cu/$N_{0.02}$C and Cu/C. **b** *k*-space. To further explore the N-doped that affect the CO_2RR activity, the XANES and FT-EXAFS of Cu/$N_{0.14}$C, Cu/$N_{0.11}$C, Cu/$N_{0.02}$C as well as Cu/C without the N-doping were plot in Fig S9a, Fig S9b. The XANES of Cu/$N_{0.11}$C, Cu/$N_{0.02}$C and Cu/C unraveled that the Cu ions were reduced to metallic copper with the decreasing of mass contents ratio of nitrogen to carbon. The FT-EXAFS data also show the intensity of the peak for Cu−Cu coordination increased while the peak at 1.5 Å for Cu−N/O coordination decreased. The FT EXAFS fitting results was shown in Fig S10 and Table S2. These results revealed that the copper in Cu/$N_{0.11}$C, Cu/$N_{0.02}$C and Cu/C agglomerated into metallic particles. The sizes of metallic particles are proportional to mass contents ratio of nitrogen to carbon. In the Cu/$N_{0.14}$C, the sizes of Cu particles were so small that the particles were oxidized to CuO_x clusters. These results agreed results well with XRD and HAADF–STEM results and indicated that N-doped promotes the dispersion of CuO_x clusters in the as-synthesized Cu/$N_{0.14}$C catalyst and played a critical role in improving FE for direct CO_2-to-ethanol electrochemical conversion.

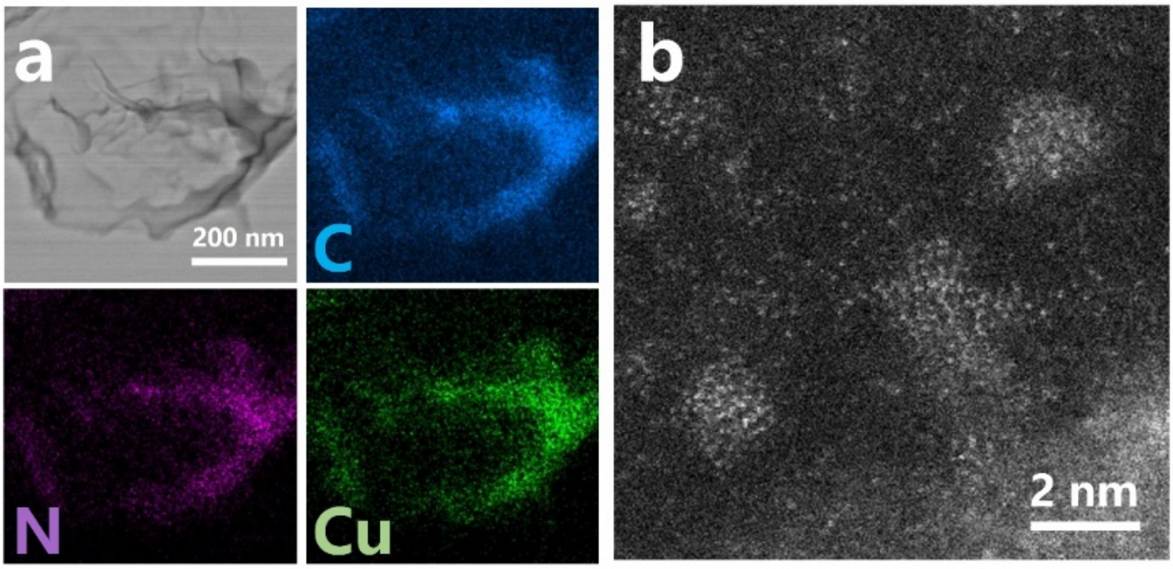

Supplementary Fig. 11 TEM image of Cu/$N_{0.14}$C after long-term CO_2RR electrolysis

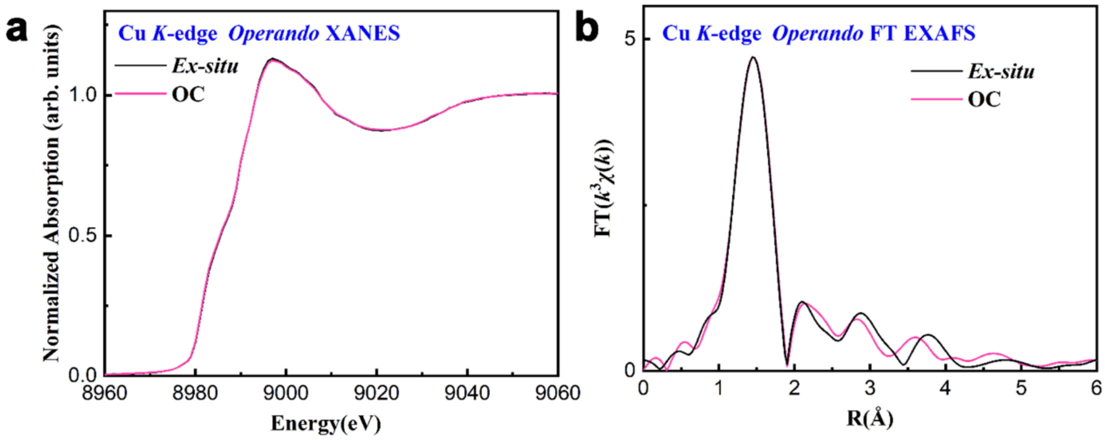

Supplementary Fig. 12 *Ex-situ* and OC XAFS characterizations of Cu/$N_{0.14}$C. a The Cu *K*-edge XANES spectra and **b** The Cu *K*-edge FT-EXAFS spectra of OC and *ex-situ* sample.

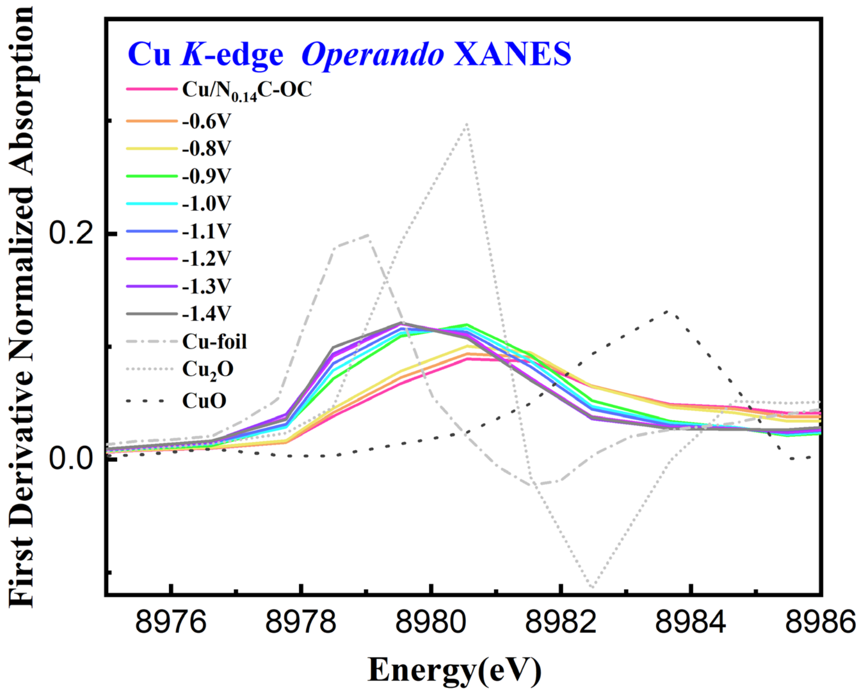

Supplementary Fig. 13 The Cu *K*-edge First derivative normalized absorption of Cu/$N_{0.14}$C at different potential.

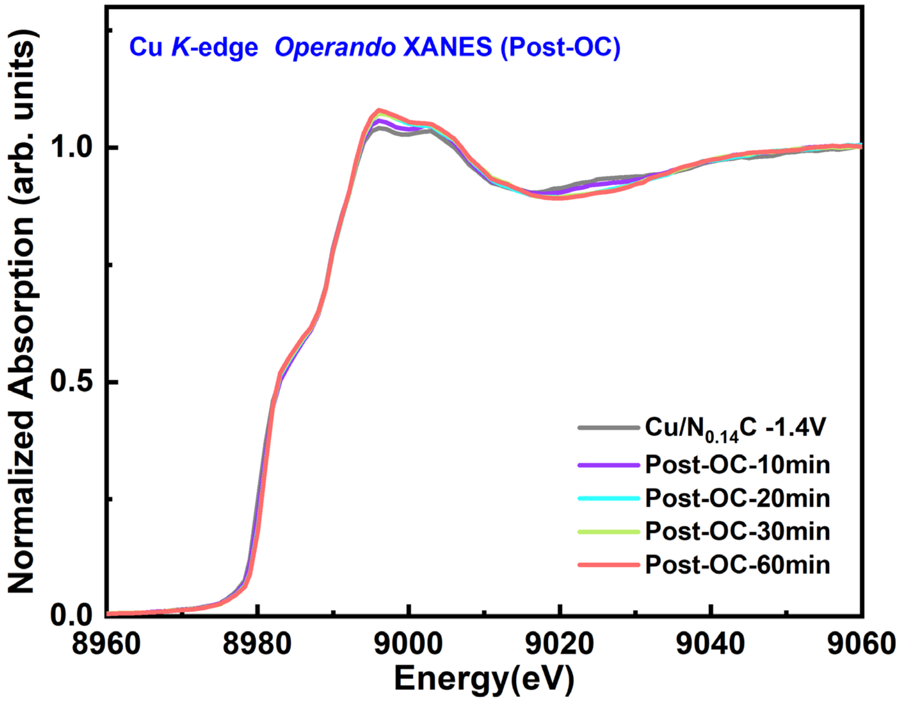

Supplementary Fig. 14 The Cu *K*-edge *operando* XANES spectra of Cu/$N_{0.14}$C after the applied potential was switched off and other conditions remained unchanged.

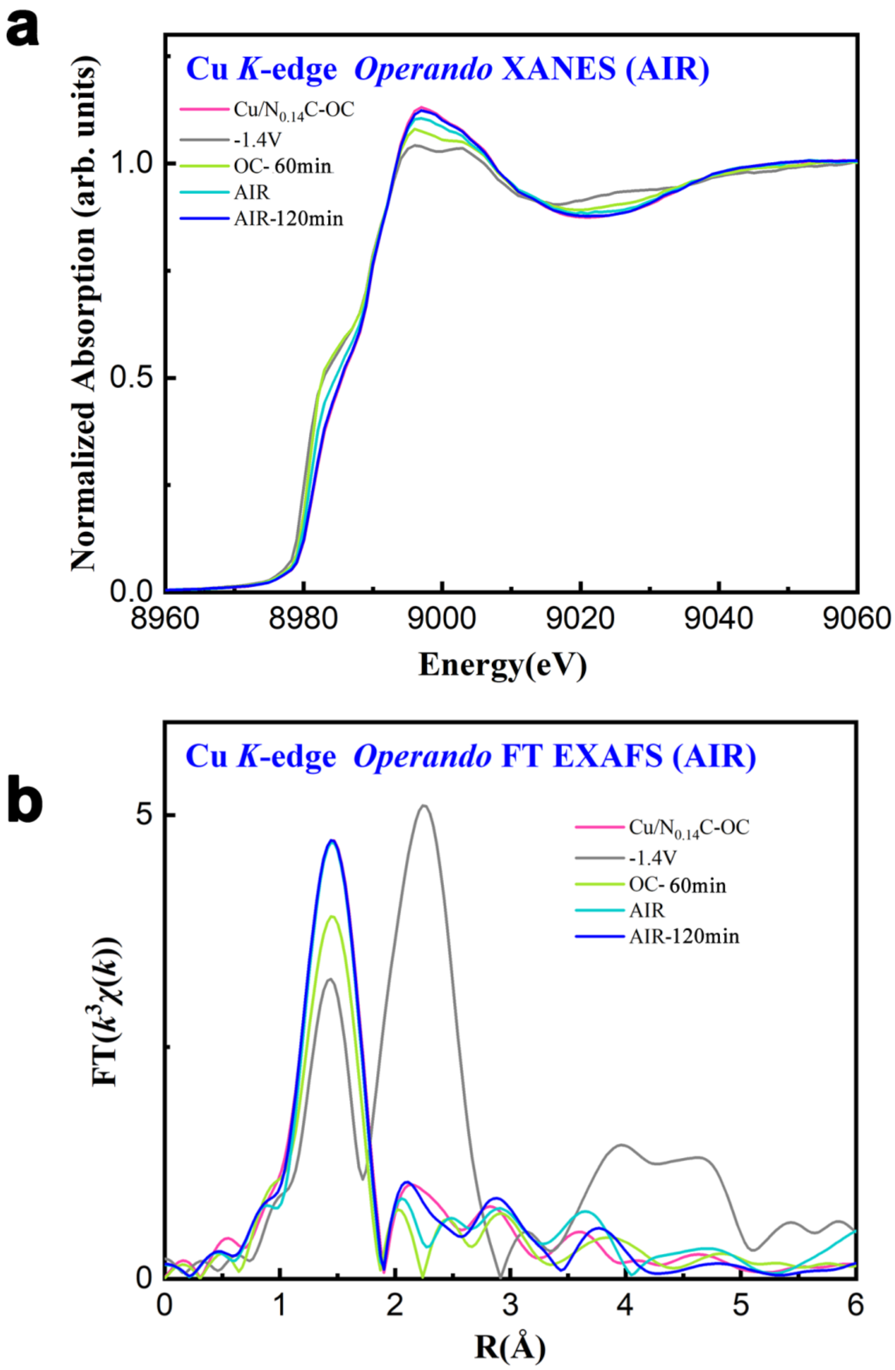

Supplementary Fig. 15 XAFS characterizations after exposure to air. a The Cu *K*-edge *operando* XANES spectra of Cu/$N_{0.14}$C-Post-OC and exposure to air. **b** *Operando* FT-EXAFS spectra of Cu/$N_{0.14}$C-Post-OC and exposure to air.

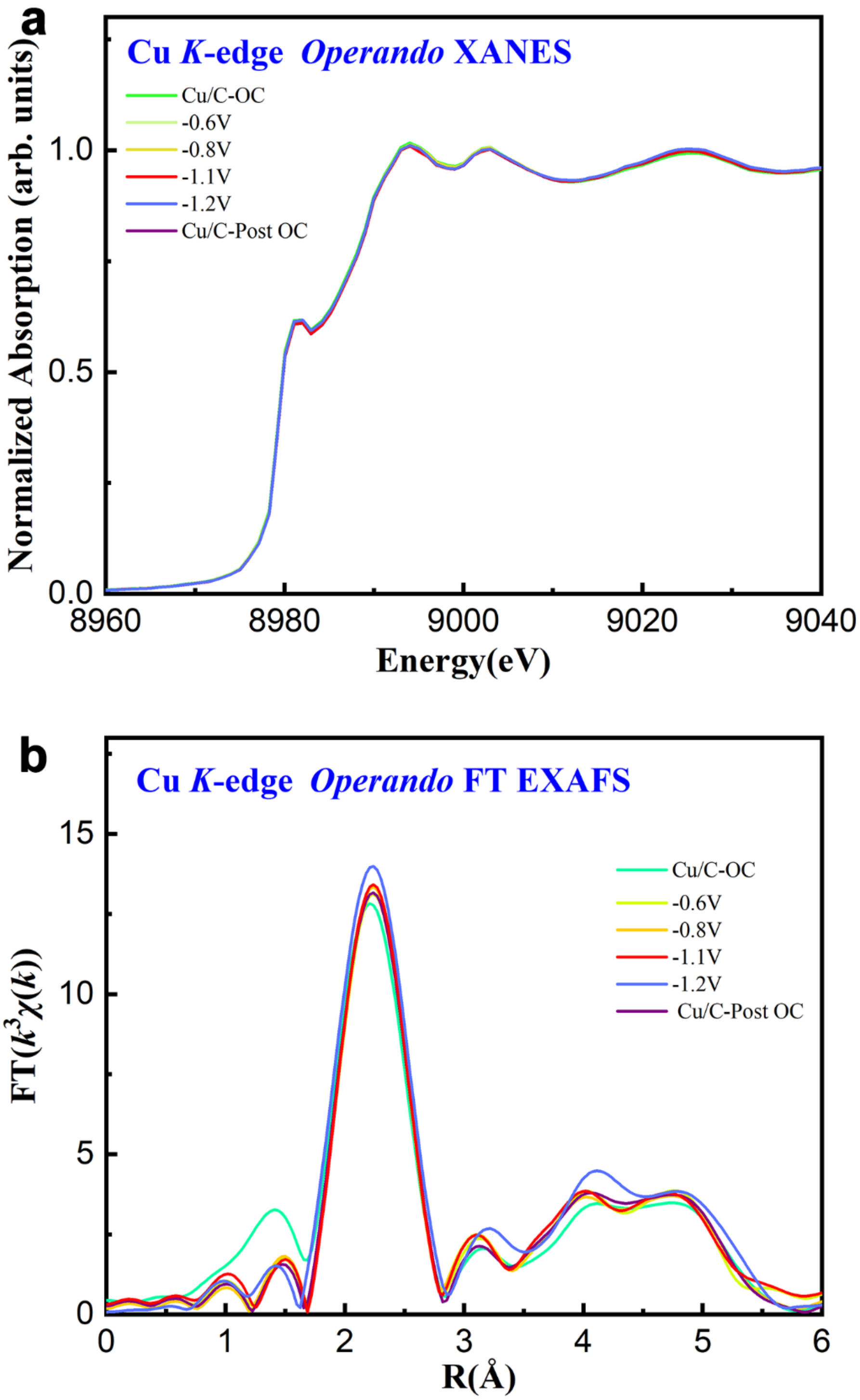

Supplementary Fig. 16 *Operando* XAFS characterization of Cu/C. a The Cu *K*-edge *operando* XANES spectra of Cu/C. **b** The Cu *K*-edge *operando* FT-EXAFS spectra of Cu/C.

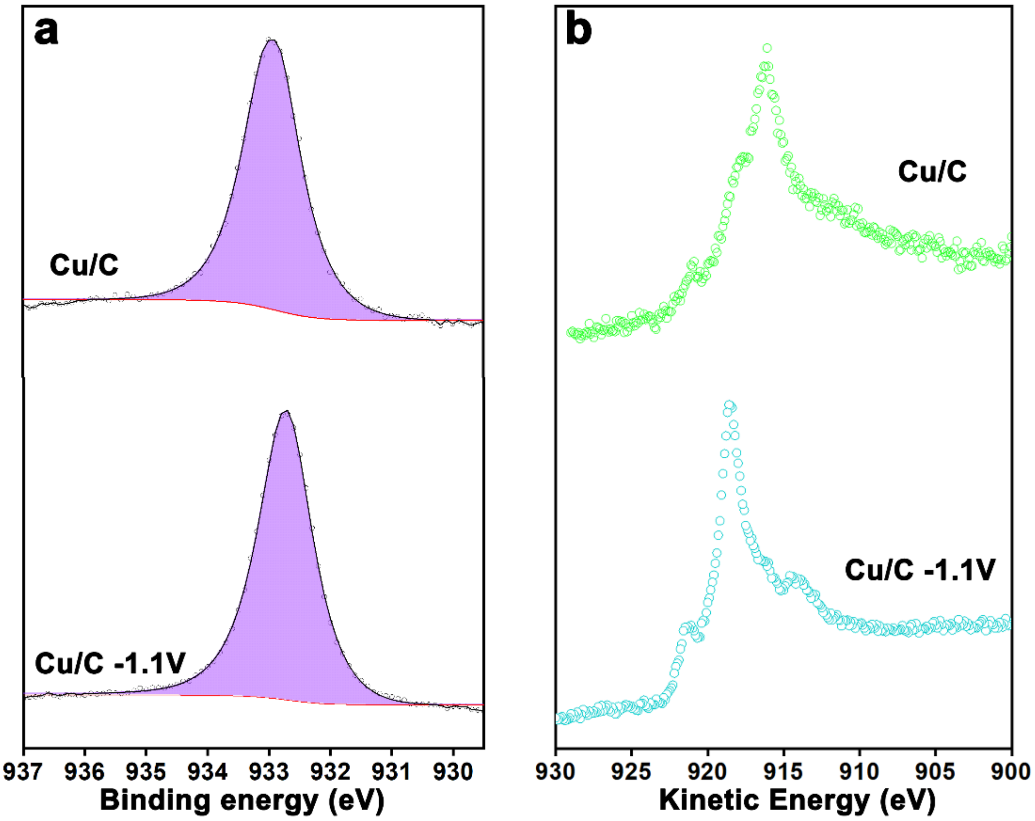

Supplementary Fig. 17 ***Quasi-in situ*** **XPS characterizations of Cu/C. a** The *Quasi-in situ* XPS spectroscopy of Cu/C. **b** The AES spectroscopy of Cu/C; All potentials are normalized to RHE.

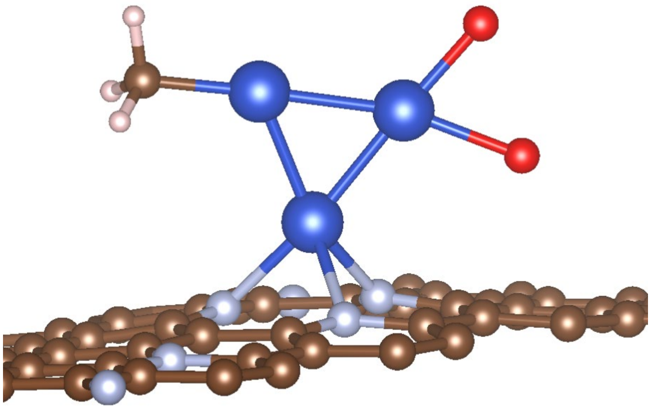

Supplementary Fig. 18 The atomic configuration of the Cu_2-CuN_3 cluster under *operando* conditions. The balls in brown, gray, red, pink and blue, represented C, N, O, H and Cu, respectively.

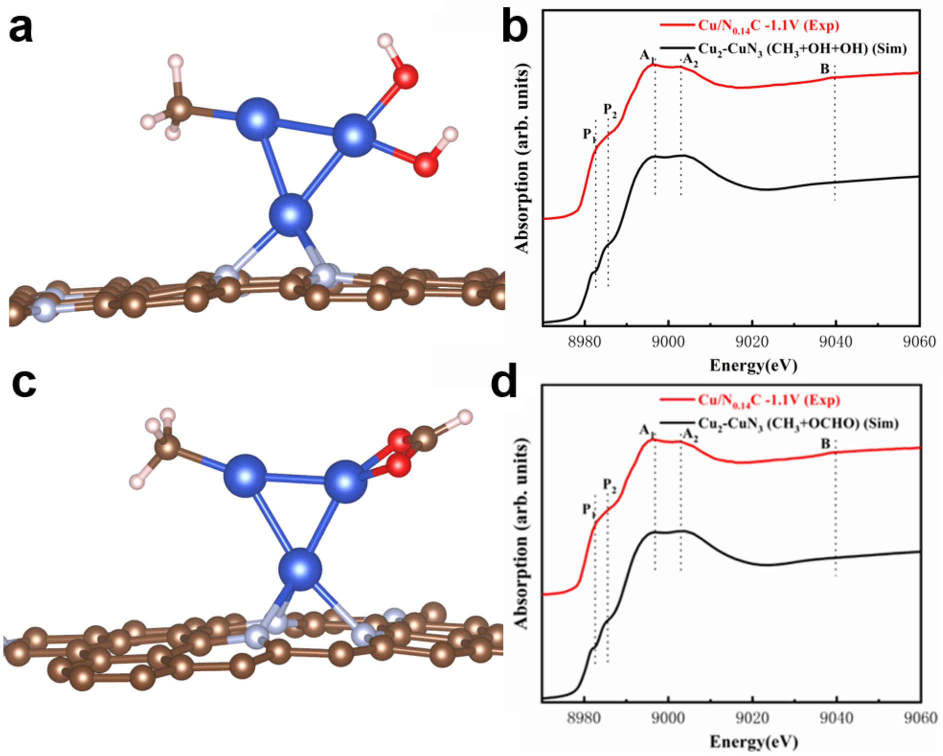

Supplementary Fig. 19 The atomic configuration of clusters and simulated XANES. a The atomic configuration of Cu_2-CuN_3 cluster with CH_3^* and two OH*. **b** Comparison between the Cu K-edge XANES experimental spectrum (black line) and the theoretical spectrum (red line) simulated with the Cu_2-CuN_3 cluster with CH_3^* and two OH*. **c** Cu_2-CuN_3 cluster with OCHO* and CH_3^*. **d** Comparison between *operando* XANES (red line) and the theoretical spectrum (black line) for the Cu_2-CuN_3 cluster with OCHO* and CH_3^*. The balls in brown, gray, red, pink and blue, represented C, N, O, H and Cu, respectively. The dotted lines corresponding to spectral XANES features were added to facilitate comparison.

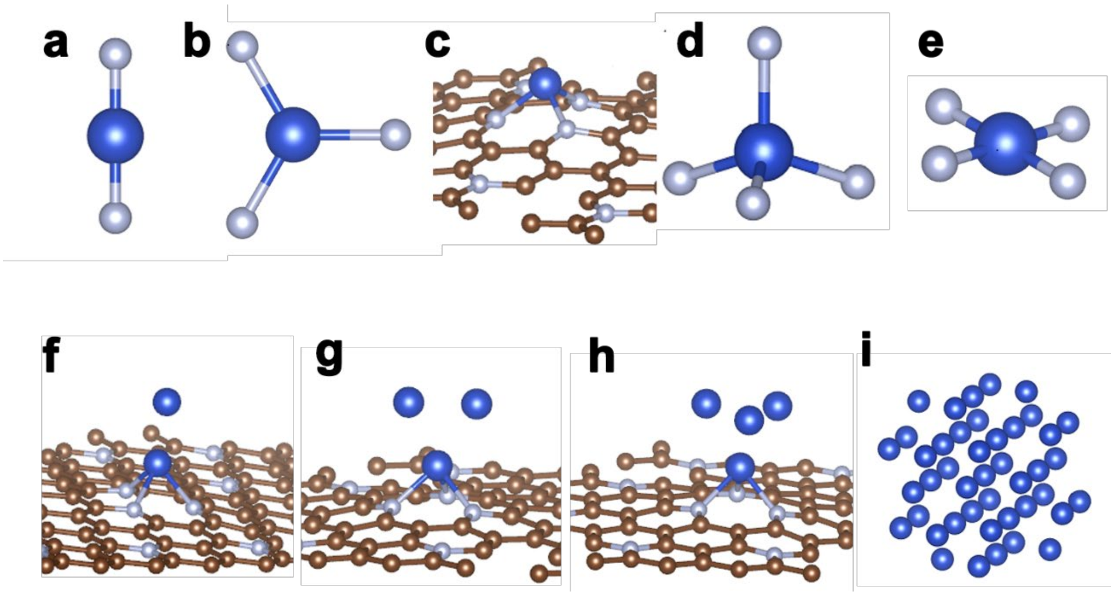

Supplementary Fig. 20 Possible sites according with the fitting results of *operando* XAFS. Cu, C and N atoms are in blue, brown and grey, respectively. *Operando* XAFS and *Quasi-in situ* XPS results revealed that the Cu^{2+} was reduced to Cu^{+} species at the -1.1 V vs. RHE. The coordination number of Cu^{+} is generally either 2, 3 or 4[2]. Therefore, there are nine possible structures of the *in situ* generated Cu species of $Cu/N_{0.14}C$ at -1.1 V vs. RHE, according with the fitting coordination number of Cu–N and Cu-Cu. **a** Cu-N_2 site with linear geometry, **b** Cu-N_3 sites with trigonal planar and **c** triangle pyramid geometry respectively, Cu-N_4 sites with **d** tetrahedral and **e** square planar geometry respectively, **f** Cu-CuN_3 site, **g** Cu_2-CuN_3 site, **h** Cu_n-CuN_3(n>2) site and **i** Cu nanoparticles.

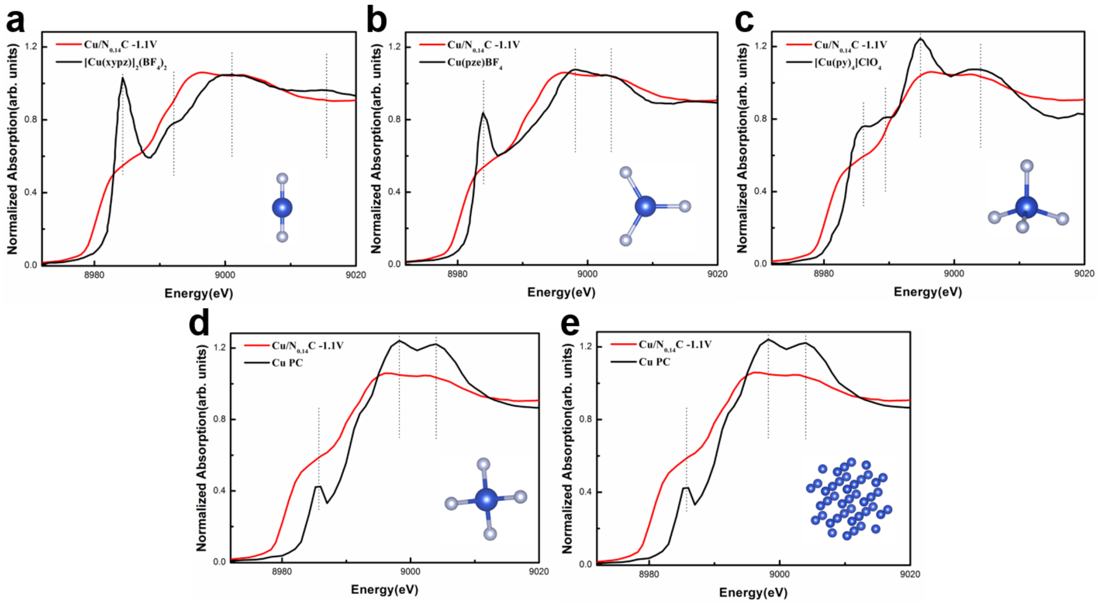

Supplementary Fig. 21 XANES of possible sites. a Cu-N_2 site, **b** Cu-N_3 site with trigonal planar geometry, **c** Cu-N_4 sites with tetrahedral and **d** square planar geometry respectively, and **e** Cu nanoparticles with the Cu $N_{0.14}C$ at -1.1 V vs. RHE. The XANES spectra shape can be more sensitive and contains more data about the material, such as the geometry around the absorbing atom and its electronic structure[3]. XANES spectra of standard samples with similar local structure around Cu ions of Cu-N_2, Cu-N_3 site with trigonal planar geometry, Cu-N_4 sites and Cu nanoparticles were measured to compare with *operando* XAFS experiment of the Cu $N_{0.14}C$ at -1.1 V vs. RHE[4]. The features of Cu-N_2, Cu-N_3 site with trigonal planar geometry, Cu-N_4 sites and Cu nanoparticles XANES spectra are notable different from that of *operando* XANES spectra at -1.1 V vs. RHE, indicating that these structures could be ruled out.

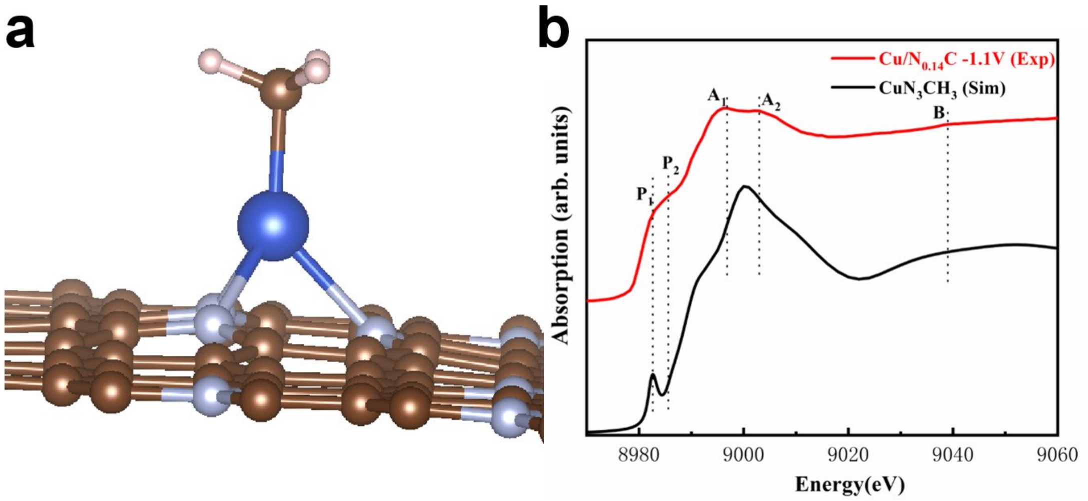

Supplementary Fig. 22 The atomic configuration of cluster and simulated XANES of CuN_3CH_3. a The atomic configuration of CuN_3CH_3. The balls in brown, gray, red, pink and blue, represented C, N, O, H and Cu, respectively. **b** Comparison between the Cu K-edge XANES experimental spectra (black line) and the theoretical spectra (red line).

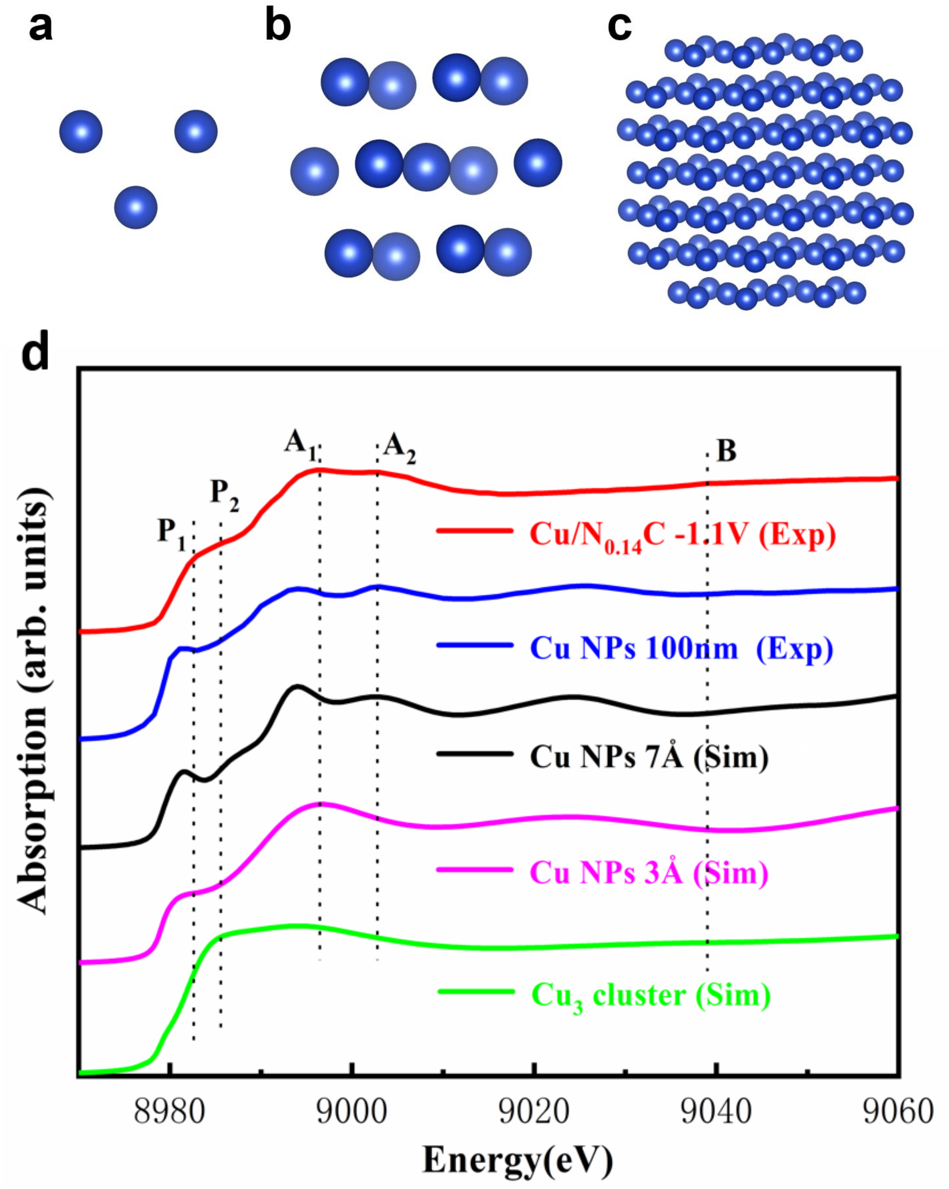

Supplementary Fig. 23 The atomic configuration of cluster and simulated XANES of different size Cu NPs clusters. **a** The atomic configuration of Cu_3. **b** 13 atoms cluster (R=3Å). **c** 135 atoms cluster (R=7Å). **d** Experimental XANES of 100 nm Cu NPs and theoretical spectra of Cu_3 cluster, 13 atoms cluster (R=3Å), 135 atoms cluster (R=7Å) with the Cu/$N_{0.14}$C at -1.1 V vs. RHE. The dotted lines corresponding to spectral XANES features were added to facilitate comparison.

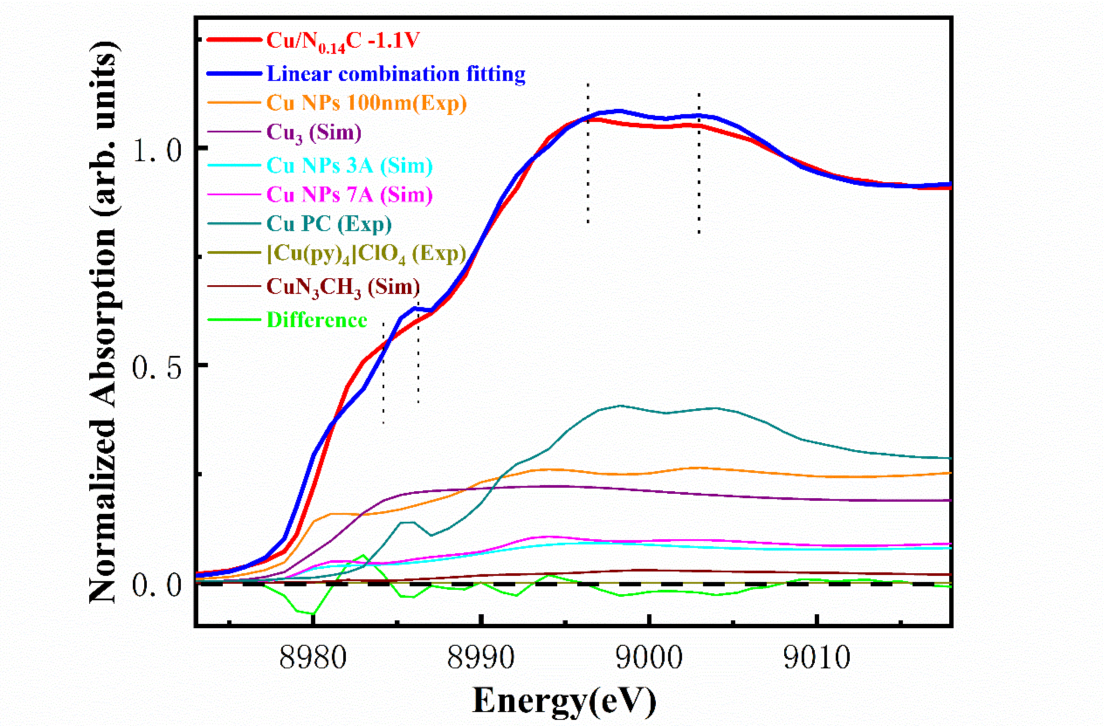

Supplementary Fig. 24 The linear combination fitting of the experimental spectrum of the Cu/$N_{0.14}$C at -1.1 V vs. RHE with the simulated spectra for CuN_3-CH_3 and Cu NPs (Cu_3 cluster, 3 Å cluster, and 7Å cluster) and the experimental spectra for the $[Cu(py)_4]ClO_4$ (Cu-N_4 site with the tetrahedral geometry) , copper porphyrin (Cu-N_4 site with the square planar geometry) and 100nm Cu NPs.

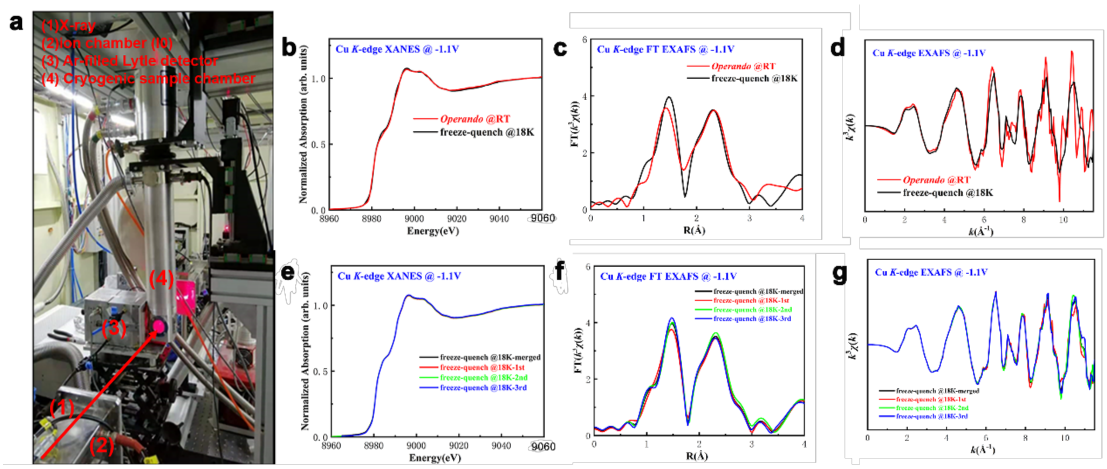

Supplementary Fig. 25 Freeze-quench XAS. a *In-house* designed cell for XAS test at 18 K. **b** Comparison between XANES of the Cu/$N_{0.14}$C obtained by the freeze-quench method (black line) and *operando* method for -1.1 V vs. RHE at room temperature (red line). **c** FT-EXAFS with the $k^3\chi(k)$. **d** $k^3\chi(k)$. **e** Comparison of three times XANES by the freeze-quench method with the merged data (black line). **f** FT-EXAFS with the $k^3\chi(k)$. **g** $k^3\chi(k)$.

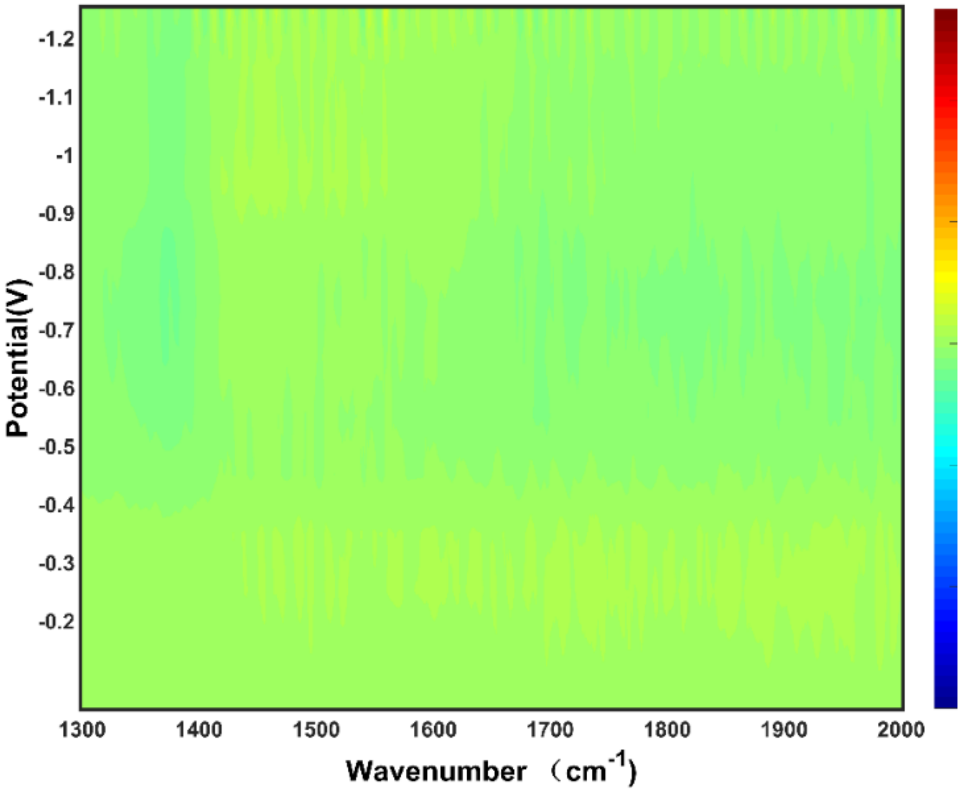

Supplementary Fig. 26 *Operando* FTIR spectroscopy for Cu/C. Potentials were normalized to RHE. The infrared bands over the Cu/C catalyst were not obviously observed even after applying potential at -1.2 V vs. RHE. These results were consistent with the low C_{2+} formation rates of the Cu/C catalyst.

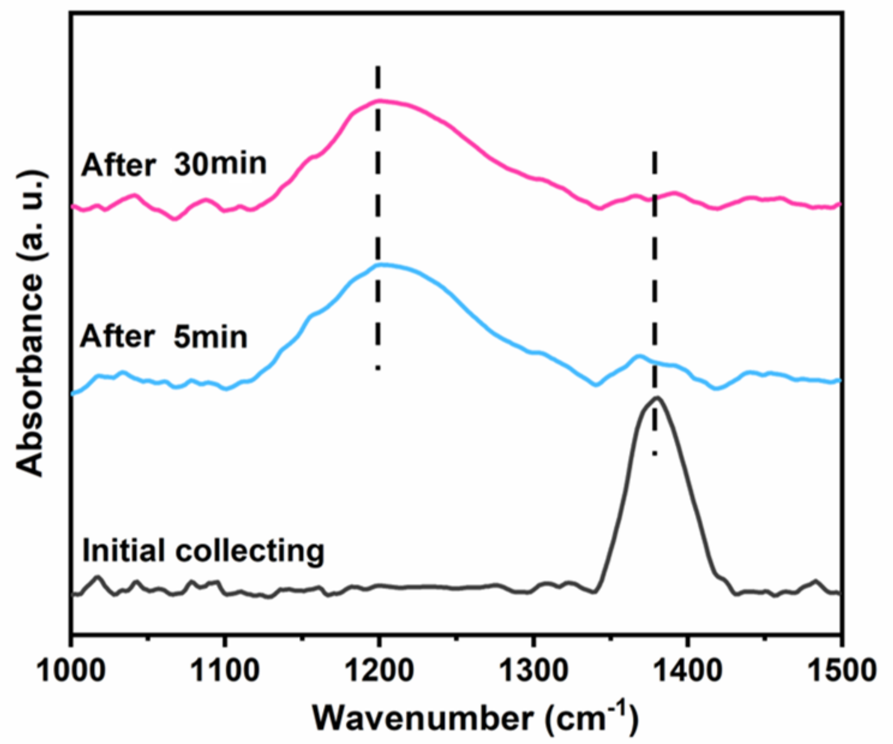

Supplementary Fig. 27 *Operando* SR-FTIR of Cu/$N_{0.14}$C in the electrolyte of D_2O.

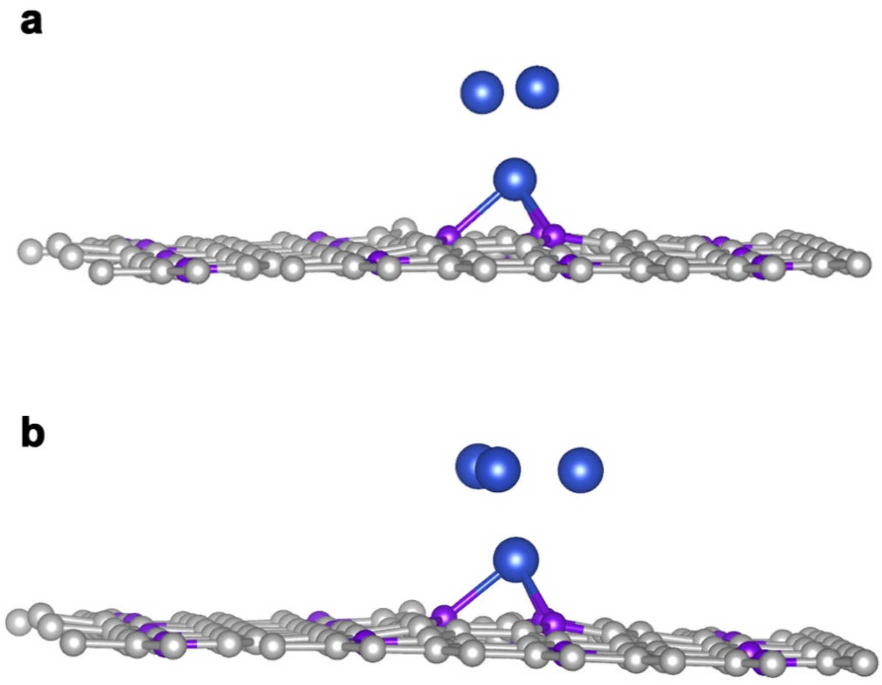

Supplementary Fig. 28 The atomic configurations. a Cu_2-CuN_3, **b** Cu_3-CuN_3. The balls in gray, purple, blue represented C, N, Cu, respectively.

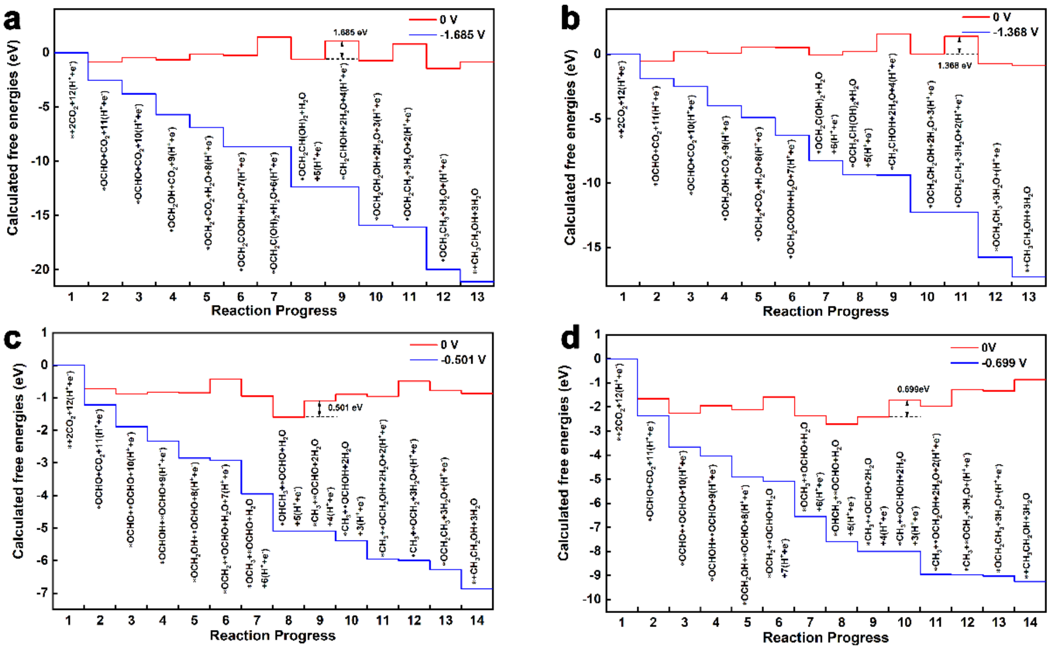

Supplementary Fig. 29 DFT calculated of CO_2RR activity. a Reaction progress of CO_2 to CH_3CH_2OH on the CuN_3 at 0 V and −1.685 V applied potential. **b** Cu-CuN_3 at 0 and −1.368 V applied potential, **c** Cu_2-CuN_3 at 0 and −0.501 V applied potential and **d** Cu_3-CuN_3 at 0 and −0.699 V applied potential. The * showed the reaction site.

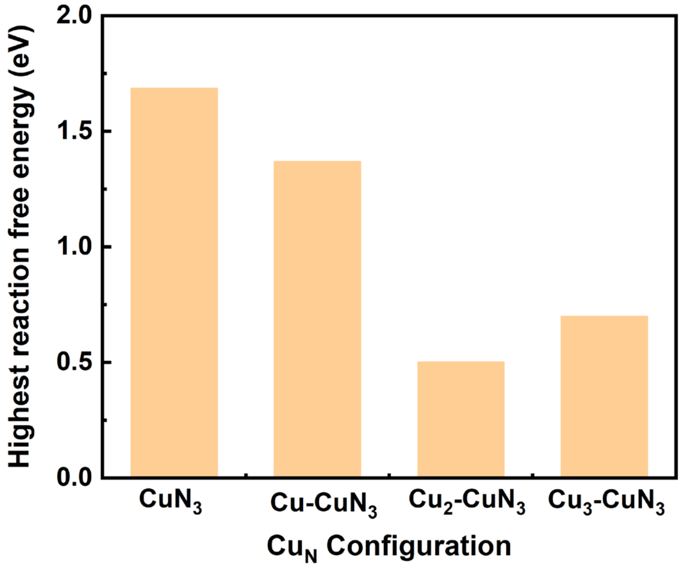

Supplementary Fig. 30 The highest reaction free energy of the Cu-N_3, Cu-CuN_3, Cu_2-CuN_3 and Cu_3-CuN_3.

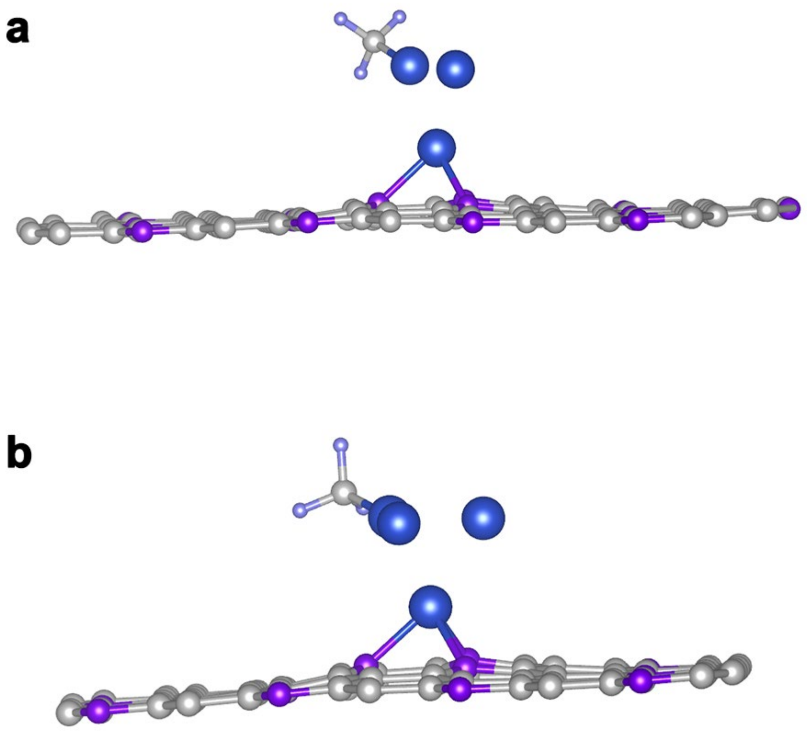

Supplementary Fig. 31 The atomic configuration of CH_3 adsorbs to Cu_n-CuN_3. a Cu_2-CuN_3. **b** Cu_3-CuN_3. Balls in gray, purple, blue and white represented C, N, Cu and H atoms, respectively.

Supplementary Table 1. Structural parameters of Cu/$N_{0.14}$C electrode from OC to -1.4 V vs. RHE in 0.1 M $KHCO_3$ as well as reference samples obtained from the Cu *K*-edge EXAFS fitting. (S_0^2=0.8) [a)]

**Sample**	**Atomic scatter**	**No. of atoms (CN)** [b)]	**Interatomic distance (Å)** [c)]	**Debye-Waller factor (10^{-3}×$Å^2$)** [d)]	**ΔE_0 (eV)** [e)]	***R* factor**
Cu-foil	Cu-Cu	12	2.54±0.03	9.1±1.8	2.2	0.001
Cu_2O	Cu-O	2	1.85±0.02	2.9±0.5	8.2	0.007
CuO	Cu-O	4	1.95±0.02	3.8±0.8	-0.4	0.003
Cu/$N_{0.14}$C	Cu-O/N	3.4±0.7	1.93±0.02	7.8±1.6	2.7	0.002
OC	Cu-O/N	3.4±0.7	1.93±0.02	7.7±1.6	2.9	0.001
-0.6V	Cu-O/N	3.3±0.6	1.92±0.02	7.0±1.4	2.5	0.001
-0.8V	Cu-O/N	2.7±0.5	1.92±0.02	6.1±1.2	2.7	0.001
	Cu-Cu	0.2±0.04	2.58±0.03	7.0±1.4	8.5	
-0.9V	Cu-O/N	2.4±0.5	1.92±0.02	6.4±1.2	2.4	0.008
	Cu-Cu	1.1±0.2	2.54±0.03	6.7±1.5	4.6	
-1.0V	Cu-O/N	2.3±0.5	1.92±0.02	6.4±1.2	2.4	0.007
	Cu-Cu	1.6±0.3	2.54±0.03	8.0±1.6	4.4	
-1.1V	Cu-O/N	2.3±0.5	1.92±0.02	7.6±1.5	2.3	0.003
	Cu-Cu	2.0±0.4	2.53±0.03	8.2±1.7	3.4	
-1.2V	Cu-O/N	2.2±0.4	1.92±0.02	9.3±1.9	2.4	0.001
	Cu-Cu	2.6±0.5	2.53±0.03	10.1±2.1	2.1	
-1.3V	Cu-O/N	2.0±0.4	1.91±0.02	6.3±1.2	1.3	0.001
	Cu-Cu	2.7±0.5	2.53±0.03	8.4±1.7	1.8	
-1.4V	Cu-O/N	2.0±0.4	1.91±0.02	7.9±1.6	1.2	0.001

	Cu-Cu	2.7±0.5	2.53±0.03	7.6±1.5	2.1	

a) S_0^2 was fixed as 0.8 based on Cu foil fitting result. Error bounds were estimated as N ± 20%.

b) CN was the coordination number.

c) Interatomic distance (R) was bond length between copper atoms and coordination atoms.

d) Debye-Waller factor (σ^2) was the static and thermal disorder.

e) ΔE_0 was the energy shift between zero kinetic energy values of the theoretical model and that of samples.

Supplementary Table 2. Structural parameters of Cu/$N_{0.14}$C as well as Cu/$N_{0.11}$C, Cu/$N_{0.02}$C and Cu/ C obtained from the Cu *K*-edge EXAFS fitting. (S_0^2=0.8). [a)]

Sample	Atomic scatter	No. of atoms (CN) [b)]	Interatomic distance (Å) [c)]	Debye-Waller factor (10^{-3}×$Å^2$) [d)]	ΔE_0 (eV) [e)]	*R* factor
Cu-foil	Cu-Cu	12	2.54±0.03	9.1±1.8	2.2	0.001
Cu/$N_{0.14}$C	Cu-O/N	3.4±0.7	1.93±0.02	7.8±1.6	2.7	0.002
Cu/$N_{0.11}$C	Cu-O/N	1.8±0.4	1.91±0.02	7.3±1.3	4.7	0.001
	Cu-Cu	4.0±0.8	2.54±0.03	7.7±1.5	3.8	
Cu/$N_{0.02}$C	Cu-Cu	6.9±1.4	2.54±0.03	8.8±1.7	3.3	0.003
Cu/C	Cu-Cu	7.3±1.5	2.54±0.03	9.0±1.8	2.1	0.013

[a)] S_0^2 was fixed as 0.8 based on Cu foil fitting result. Error bounds were estimated as N ± 20%.

[b)] CN was the coordination number.

[c)] Interatomic distance (R) was bond length between copper atoms and coordination atoms.

[d)] Debye-Waller factor (σ^2) was the static and thermal disorder.

[e)] ΔE_0 was the energy shift between zero kinetic energy values of the theoretical model and that of samples.

Supplementary Table 3. Comparison of CO_2RR activities for some catalysts.

No.	Catalyst	Electrolyte	Potential (V vs. RHE)	Partial current density (mA cm^{-2})	Product	FE %	C_{2+} FE %	Electrode	Ref.
	$Cu/N_{0.14}C$	0.1 M $KHCO_3$	-1.1	14.4	C_2H_5OH	51	73	**H-type Cell(HC)**	**This work**
1	Cu–P1	1 M KOH	-0.97	312	C_2H_4	72	90	Flow Cell (FC)	*Nat. Catal.*, **2021**, 4, 20-27
		10 M KOH	-0.47	-		87	93		
2	Cu/GDL	2 M KCl + 0.01 M KHCO3	-1.2	-	C_2H_4	39.9	82	HC	*J. Am. Chem. Soc.* **2021,** 143, 3245-3255
3	20% Cu/CuSiO3	0.1 M $KHCO_3$	-1.1	20.20	C_2H_4	51.8	-	HC	*Angew. Chem. Int. Ed.*, **2021,** 60, 15344-15347
4	34% N-C/Cu	1 M KOH	-0.68	156	C_2H_5OH	52	93	FC	*Nat. Energy,* **2020**, 5, 478-486
5	$Cu-CO_2$	7 M KOH	-0.67	280	C_2H_4	60	90	FC	*Nat. Catal.,* **2020**, 3， 98-106
6	Cu-SA/NPC	0.1 M $KHCO_3$	-0.36	-	CH_3COCH_3	36.7	-	HC	*Nat. Commun.* **2020**, *11*, 2455
7	OBC (Cu_4O)	0.5 M $KHCO_3$	-0.95	44.7	C_2H_4	45	-	HC	*J. Am. Chem. Soc.* **2020**, 142, 26, 11417–11427
8	H-Cu MPs	0.1 M $KHCO_3$	-0.82	9	C_2H_4	50.1	67.3	HC	*Nano Letters*, **2020**, 20, 7, 4823–4828
9	CuSAs/TCNFs	0.1 M $KHCO_3$	-0.9	93	CH_3OH	44	-	HC	*J. Am. Chem. Soc.* **2019**, 141, 12717−12723
10	$Cu_{0.5}NC$	0.1 M $CsHCO_3$	-1.2	12	C_2H_5OH	55	80	HC	*Angew. Chem. Int. Ed.*, **2019**, 58, 15098-15103.
11	$Ag_{0.14}/Cu_{0.86}$	1 M KOH	-0.67	250	C_2H_5OH	41	80	FC	*J. Am. Chem. Soc.*, **2019**, 141, 8584-8591
12	CuPc	0.5 M $KHCO_3$	-1.06	13	CH_4	66	-	HC	*Nat. Commun.* **2018**, *9*, 415.
13	Cu(B)-2	0.1 M KCl	-1.1	70	C_2H_4	52	79	HC	*Nat. chem.*, **2018**,10, 974-980
14	Mesopore Cu 30/40	0.1 M $KHCO_3$	-1.7	5.7	C_2H_4	38	46	HC	*Angew. Chem. Int. Ed.*, **2017**,56, 796-800

3. Supplementary References

1. Ravel, B. & Newville, M. ATHENA, ARTEMIS, HEPHAESTUS: data analysis for X-ray absorption spectroscopy using IFEFFIT. *J. of Synchrotron Radiat.* **12**, 537-541 (2005).
2. O Bunău and Y Joly. *J. Phys.: Condens. Matter* **21** 345501 (2009).
3. Kau, L. *et al.* X-ray Absorption Edge Determination of the Oxidation State and Coordination Number of Copper: Application to the type 3 site in Thus vernicifera Laccase and Its Reaction with Oxygen. *J. Am. Chem. Soc.* **109**, 6433-6442 (1987).
4. Bokhoven *et al.* X-ray Aborption and X-ray Emission Spectroscopy Theory and Applications. (Wiley, 2016).
