# Supplementary material for: Complementary Operando Spectroscopy identification of in-situ generated metastable charge-asymmetry Cu2-CuN3 clusters for CO2 reduction to ethanol
Source: Nat Commun. 2022 Mar 11;13:1322. doi: 10.1038/s41467-022-29035-8 (PMC8917205; doi:10.1038/s41467-022-29035-8)
Supplement: Supplementary file 2 — Description of Additional Supplementary Files [file 41467_2022_29035_MOESM2_ESM.docx]

**Description of Additional Supplementary Files**

File Name: Supplementary Data 1

Description: The DFT data as cif file of Cu_2_-CuN_3_

File Name: Supplementary Data 2

Description: The DFT data as cif file of Cu_2_-CuN_3_ with *OCHO

File Name: Supplementary Data 3

Description: The DFT data as cif file of Cu_2_-CuN_3_ with *OCHO + *OCHO

File Name: Supplementary Data 4

Description: The DFT data as cif file of Cu_2_-CuN_3_ with *OCHOH + *OCHO

File Name: Supplementary Data 5

Description: The DFT data as cif file of Cu_2_-CuN_3_ with *OCH_2_OH + *OCHO

File Name: Supplementary Data 6

Description: The DFT data as cif file of Cu_2_-CuN_3_ with *OCH_2_ + *OCHO

File Name: Supplementary Data 7

Description: The DFT data as cif file of Cu_2_-CuN_3_ with *OCH_3_ + *OCHO

File Name: Supplementary Data 8

Description: The DFT data as cif file of Cu_2_-CuN_3_ with *OHCH_3_ + *OCHO

File Name: Supplementary Data 9

Description: The DFT data as cif file of Cu_2_-CuN_3_ with *CH_3_ + *OCHO

File Name: Supplementary Data 10

Description: The DFT data as cif file of Cu_2_-CuN_3_ with *CH_3_ + *OCHOH

File Name: Supplementary Data 11

Description: The DFT data as cif file of Cu_2_-CuN_3_ with *CH_3_ + *OCH_2_OH

File Name: Supplementary Data 12

Description: The DFT data as cif file of Cu_2_-CuN_3_ with *CH_3_ + *OCH_2_

File Name: Supplementary Data 13

Description: The DFT data as cif file of Cu_2_-CuN_3_ with *OCH_2_CH_3_
